# Sensitivity-Based Fault Detection and Isolation Algorithm for Road Vehicle Chassis Sensors

**DOI:** 10.3390/s18082720

**Published:** 2018-08-18

**Authors:** Wonbin Na, Changwoo Park, Seokjoo Lee, Seongo Yu, Hyeongcheol Lee

**Affiliations:** 1 Department of Electrical Engineering, Hanyang University, Seoul 04763, Korea; nao6114@hanyang.ac.kr (W.N.); changwoo@hanyang.ac.kr (C.P.); 2 Chassis System Control Development Team, Hyundai Motor Company, 150 Hyundaiyeonguso-ro, Namyang-eup, Hwaseong-si 18280, Korea; setmail@hyundai.com (S.L.); soyoo@hyundai.com (S.Y.); 3 Department of Electrical and Biomedical Engineering, Hanyang University, Seoul 04763, Korea

**Keywords:** road vehicle, fault diagnosis, fault detection and isolation, sensitivity analysis, model-in-the-loop, hardware-in-the-loop

## Abstract

Vehicle control systems such as ESC (electronic stability control), MDPS (motor-driven power steering), and ECS (electronically controlled suspension) improve vehicle stability, driver comfort, and safety. Vehicle control systems such as ACC (adaptive cruise control), LKA (lane-keeping assistance), and AEB (autonomous emergency braking) have also been actively studied in recent years as functions that assist drivers to a higher level. These DASs (driver assistance systems) are implemented using vehicle sensors that observe vehicle status and send signals to the ECU (electronic control unit). Therefore, the failure of each system sensor affects the function of the system, which not only causes discomfort to the driver but also increases the risk of accidents. In this paper, we propose a new method to detect and isolate faults in a vehicle control system. The proposed method calculates the constraints and residuals of 12 systems by applying the model-based fault diagnosis method to the sensor of the chassis system. To solve the inaccuracy in detecting and isolating sensor failure, we applied residual sensitivity to a threshold that determines whether faults occur. Moreover, we applied a sensitivity analysis to the parameters semi-correlation table to derive a fault isolation table. To validate the FDI (fault detection and isolation) algorithm developed in this study, fault signals were injected and verified in the HILS (hardware-in-the-loop simulation) environment using an RCP (rapid control prototyping) device.

## 1. Introduction

The vehicle control system improves the performance of the braking, steering, and suspension. ESC (electronic stability control), which is a vehicle chassis control system, is used to maintain the driving stability in consideration of the driving situation of the driver, the vehicle condition, and the road conditions [1,2]. The steering system MDPS (motor-driven power steering) receives the steering input of the driver and provides assistant torque to the support steering [3,4]. The ECS (electronically controlled suspension) system can also maintain the stability and ride comfort by varying the height of the vehicle body depending on the road surface condition and the driving conditions [5,6,7].

An advanced driving assistance system (ADAS), which is more advanced than the traditional driving assistance system (DAS), is a system that assists drivers with advanced systems, and is the subject of many current research projects. Recently, parking assistance systems (PAS) have also been developed to accurately identify obstacles and to park a car automatically using sensor fusion [8]. Autonomous emergency braking (AEB) has also been developed to cope with emergencies that are difficult for drivers to handle [9]. Adaptive cruise control (ACC) was first proposed in the 1960s to assist drivers and, more recently, to take into account not only convenience and safety but also fuel economy to make optimal driving possible [10]. Further studies have undertaken research into lane-keeping assistance systems (LKAS) which recognize lanes through sensors and prevent lane departure [11,12,13].

Therefore, sensors in intelligent vehicles that use sensors to control cars and in autonomous vehicles that run without driver input are essential. However, contingency plans are also needed if these sensors fail. A car that operates based on sensor signals can pose a fatal threat to a car, driver, and even pedestrians when a sensor fails. For this reason, diagnosing sensor failures is an important task for researchers who develop smart cars.

**Figure 1:**
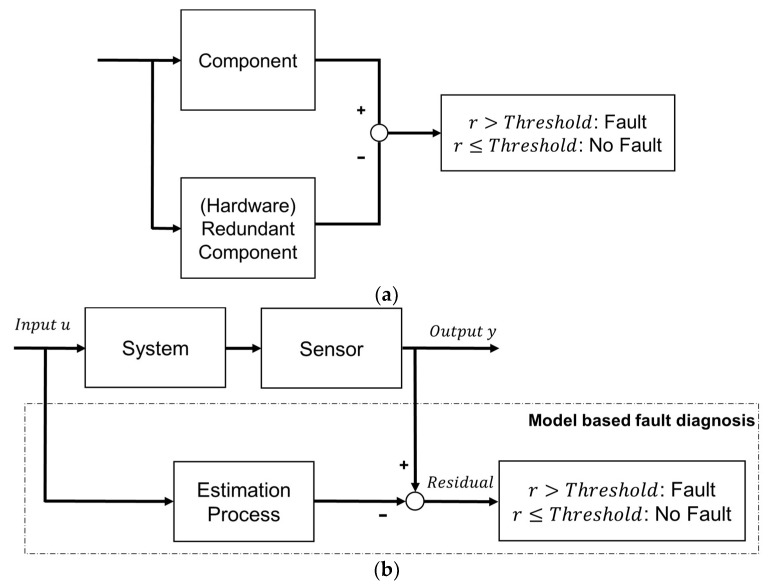
Hardware and analytical redundancy scheme. (**a**) Hardware redundancy scheme; (**b**) analytical redundancy scheme.

The fault diagnosis method can be classified into a hardware redundancy method and an analytical redundancy method [14,15,16,17]. The hardware redundancy method in Figure 1a uses two or more sensors and actuators to ensure that stable fault diagnosis and normal operation are possible and have high reliability. However, this method has a disadvantage in that cost and space for duplication are required. Therefore, an analytical redundancy method in Figure 1b using mathematical relations between signals that overcome these problems has been proposed and studied. These methods of preparing for failure by creating an analytical redundancy make it possible to diagnose failure with algorithms without investing in space and cost, and thus enabling the commercialization of smart cars.

However, there are limitations to performing fault diagnosis with multiple residuals. This is because there is a difference between a mathematical model that is considered for diagnosing faults and a real car. Researchers in past studies have called this model uncertainty. In other words, when many residuals are applied, there are model uncertainties of different sizes and types in each of the residuals. Model uncertainty can have a fatal impact on each of the analytical redundancies. For this reason, it is difficult to detect and isolate a fault using multiple residuals. In previous studies, developers have set arbitrary limits to account for this model uncertainty. However, the analytical method of fault-finding with dynamic models is problematic for model uncertainty. Therefore, an adaptive thread using the frequency of input expressions has also been studied and widely used [18].

Recently, a study was conducted on an observer using real-time fuzzy calculations to diagnose faults using neural networks [19]. Moreover, with the significant improvement in computational power and efficiency of computers, research was conducted to detect and classify faults using machine learning [20]. A further study suggested failure diagnosis using a new method, Gaussian Mixer Modeling, to apply model-based fault-finding for nonlinear systems [21]. In contrast, a randomized failure detection method was also proposed using a generalized canonical correlation analysis without the use of a Gaussian model [22]. Another groundbreaking study was also conducted to diagnose faults on systems without sensors using only dynamic models [23]. However, research has yet to be conducted on systems where the accurate determination and separation of failures are critical, with many types of sensors operating simultaneously, as is the case in automobiles.

This paper is based on a study first reported in the Korean *Journal of Transaction of KSAE* [24]. In this paper, we propose a new method of fault diagnosis, sensitivity-based fault detection, and isolation. Section 2 introduces and describes the sensitivity-based fault method. In Section 3, estimation is performed based on vehicle dynamics to diagnose faults on sensors in the vehicle and verified using Carsim, a vehicle dynamics simulator. As a next step, the residual is calculated using the proven estimation and sensor values. In Section 4, the calculated residual expression is used to calculate the sensitivity to the fault signal. In Section 5, we verify the sensitivity-based FDI (fault detection and isolation) algorithm introduced in Section 2. For verification, we inject the failure of each sensor into the simulation environment based on HILS (hardware-in-the-loop simulation) and examine the results.

## 2. Fault Detection and Isolation Algorithm

### 2.1. Residual Generation, Threshold Review

**Figure 2:**
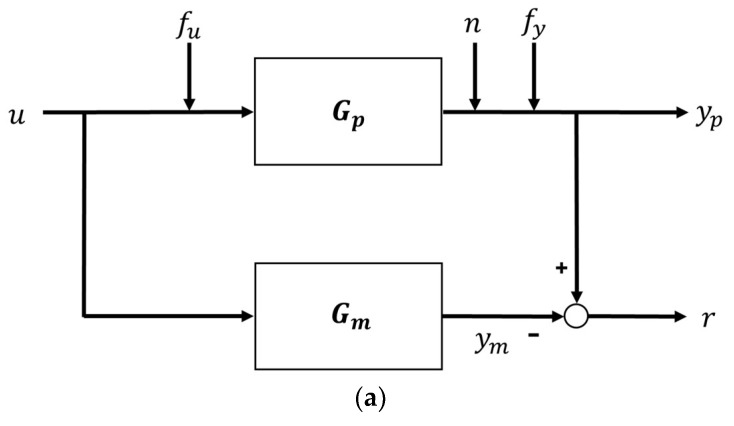

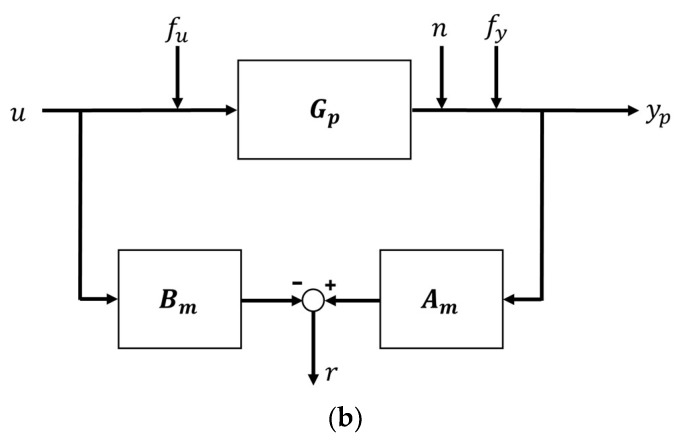
Residual calculation with output error and polynomial method. (**a**) Output error method residual calculation; (**b**) polynomial error method residual calculation.

The method for generating the residual is shown in Figure 2. It shows how to use the output error method and polynomial error method using the input and output models of the system.

(1) $$r=y_{p}-y_{m}\;\left(Output\;error\right)$$

(2) $$r=A_{m}\left(s\right)y_{p}-B_{m}\left(s\right)u\;\left(Polynomial\;error\right)$$

If the model is correct, the residual would be zero for normal conditions, and it would be non-zero when a failure exists. However, there is the model uncertainty due to the system, which is estimated by the model. To solve this model uncertainty, the existing model-based fault diagnosis is based on the following fault detection conditions.

(3) $$r\left(\Delta \right)>TH_{\Delta }\;\left(Fault\;detection\;condition\right)$$

(4) $$r\left(\Delta \right)\leq TH_{\Delta }\;\left(No\;Fault\;detection\;condition\right)$$

Unlike Equations (3) and (4), which are set threshold targets based on the residual, the new condition is calculated to the fault signal expressed in Equation (5).

### 2.2. Sensitivity Applied Fault Detection and Isolation

(5) $$f\left(t\right)>TH_{f}$$

where f represents the fault signal and $TH_{f}$ indicates the maximum allowable range of the fault. In this case, we can rearrange the threshold value based on the residual value, which is the actual result of the algorithm for detecting the fault. Therefore, the fault signal can be expressed as a combination of the residual sensitivity of the fault signal and the residual of the fault signal, as shown in Equations (6) and (7).

(6) $$r\left(f\left(t\right)\right)=\frac{\partial r}{\partial f}f\left(t\right)$$

(7) $$f\left(t\right)=\frac{\partial r}{\partial f}r\left(f\left(t\right)\right)>TH_{f}$$

Therefore, the fault detection condition can be defined by Equation (8) and, finally, the fault detection condition of Equation (9) can be derived for the threshold considering model uncertainty [24].

(8) $$r\left(f\left(t\right)\right)>\frac{\partial r}{\partial f}TH_{f}$$

(9) $$r\left(\Delta ,\;f\left(t\right)\right)>TH_{\Delta }+\frac{\partial r}{\partial f}TH_{f}$$

**Figure 3:**
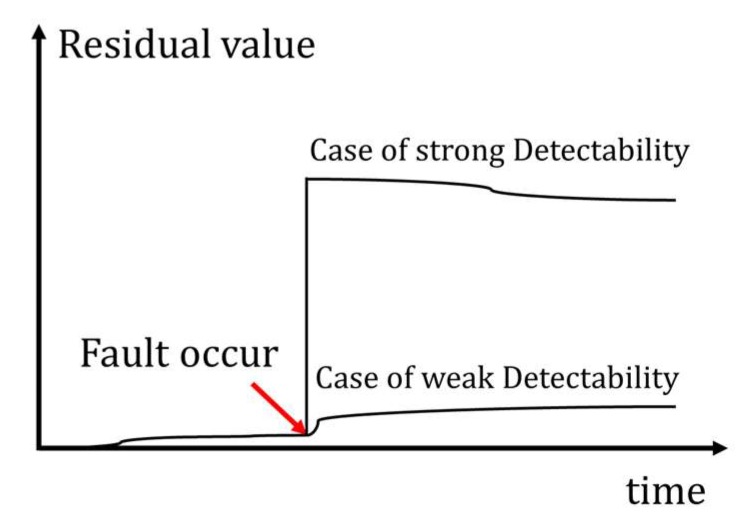
Fault detectability (strong/weak).

In previous fault diagnosis studies, the concept of fault detectability has been presented as in Figure 3 [19]. In this paper, the detectability of the fault is considered using residual sensitivity. For the fault isolation, the sensitivity of each residual equation is analyzed, and the fault isolation table is also derived from the analysis of residual equations for the fault detectability.

**Figure 4:**
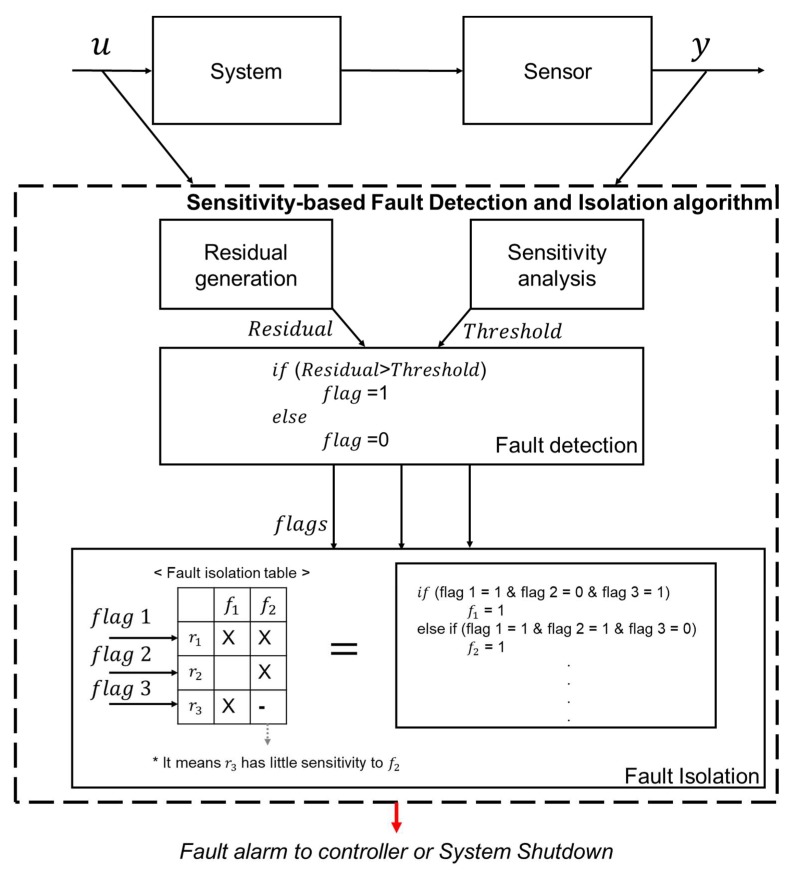
Sensitivity-based fault detection and isolation algorithm scheme.

Finally, the model-based residual value and fault detection condition are calculated in real time for fault diagnosis. These were applied to the fault detection and isolation algorithm as shown in Figure 4. This algorithm compares the residual with a threshold value and generates a fault flag, indicating that the corresponding residual is abnormal. Considering residual sensitivity as detectability, we can separate faults using these flags.

## 3. Vehicle Dynamics-Based Residual Generation and Simulation

We used constraints based on vehicle dynamics to diagnose faults in sensors used in automobiles. For the convenience of developing the FDI algorithm, we calculated the residuals of the wheel angular speed, steering wheel angle, and normal force of each wheel. To estimate and generate the roll angle residual, we used the polynomial error method and verified the algorithm.

In Section 3, we estimate and simulate the wheel speed and steering wheel angle output of sensors in the vehicle to calculate the residual. However, estimates without a sensor, such as normal force and roll angle, are also addressed by the FDI algorithm, so the overall estimate was validated using Carsim (a vehicle dynamics simulator). This study assumes a single fault. Thus, it was assumed that robust estimation of longitudinal speed is possible by using other speed and acceleration sensors [25,26]. It was also assumed that the car was traveling on a level surface without bank angle and grade, the most common road type.

### 3.1. Wheel Angular Speed Residual

(10) $${\hat{v}}_{fl}=v_{x}-\dot{\psi }\left(\frac{l_{tw}}{2}-l_{f}\frac{v_{y}}{v_{x}}\right)$$

(11) $${\hat{v}}_{fr}=v_{x}+\dot{\psi }\left(\frac{l_{tw}}{2}+l_{f}\frac{v_{y}}{v_{x}}\right)$$

(12) $${\hat{v}}_{rl}=v_{x}-\frac{\dot{\psi }l_{tw}}{2}$$

(13) $${\hat{v}}_{rr}=v_{x}+\frac{\dot{\psi }l_{tw}}{2}$$

**Figure 5:**
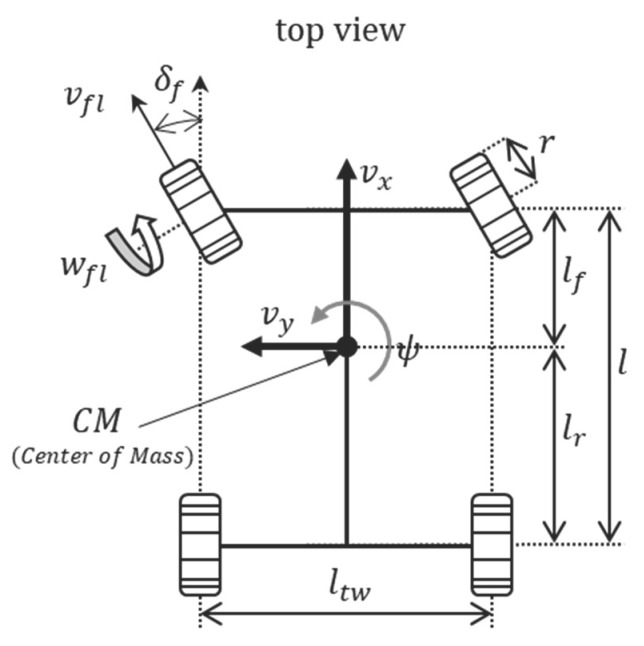
Vehicle wheel scheme.

Figure 5 shows vehicle wheel with the mass center of the vehicle. Equations (10) and (11) are equations for the front left and right wheel speeds, where $v_{x}$ is the longitudinal speed of the vehicle, $\dot{\psi }$ is the yaw rate, $l_{tw}$ is the track width, $l_{f}$ is the length of the wheel base, $v_{y}$ is the lateral speed of the vehicle, $v_{fl}$ is the longitudinal speed of the front left wheel, $v_{fr}$ is the longitudinal speed of the front right wheel, $v_{rl}$ is the longitudinal speed of the rear left wheel, and $v_{rr}$ is the longitudinal speed of the rear right wheel [27,28].

**Figure 6:**
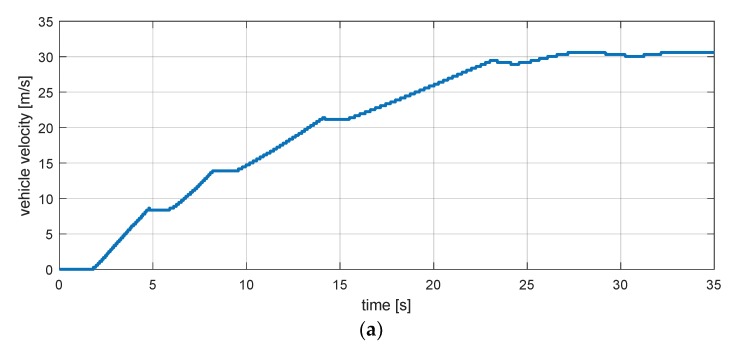

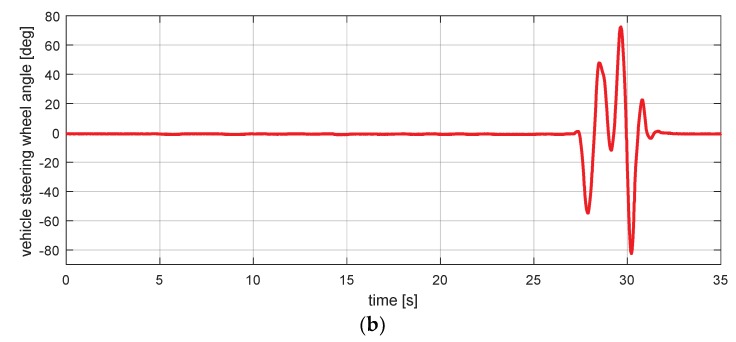
Estimation simulation scenario 1. (**a**) Vehicle speed; (**b**) steering wheel angle.

**Figure 7:**
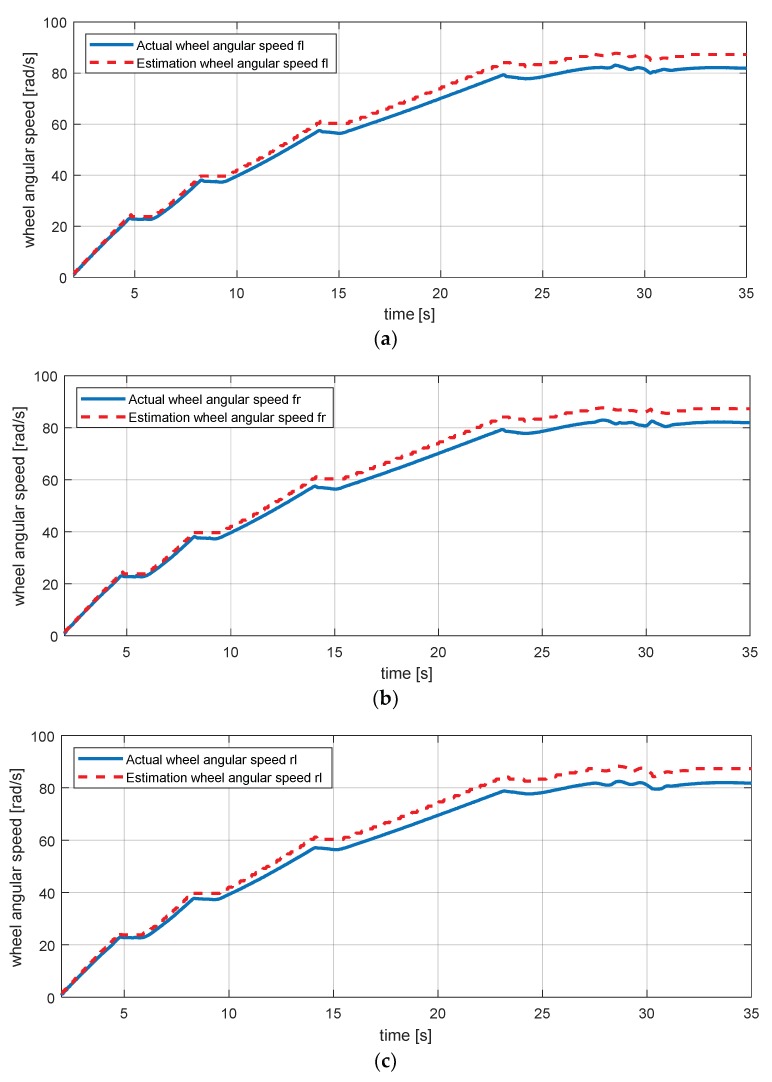

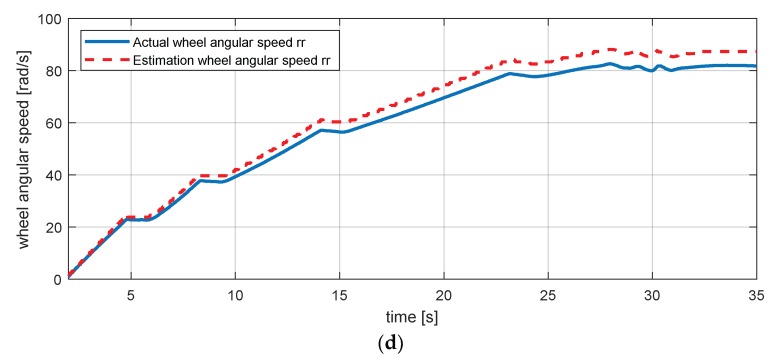
Estimation simulation result for wheel angular speed. (**a**) Wheel angular speed—fl (front left); (**b**) wheel angular speed—fr (front right); (**c**) wheel angular speed—rl (rear left); (**d**) wheel angular speed—rr (rear right).

Equations (12) and (13) are equations for the rear left and right wheel speeds. To validate the dynamic equation, the simulation scenario in Figure 6 was used [29,30]. Results of the simulation in Figure 7 show that minor errors exist as the vehicle speed increases. In other words, we can see that there is a model uncertainty that occurs when a vehicle accelerates.

**Figure 8:**
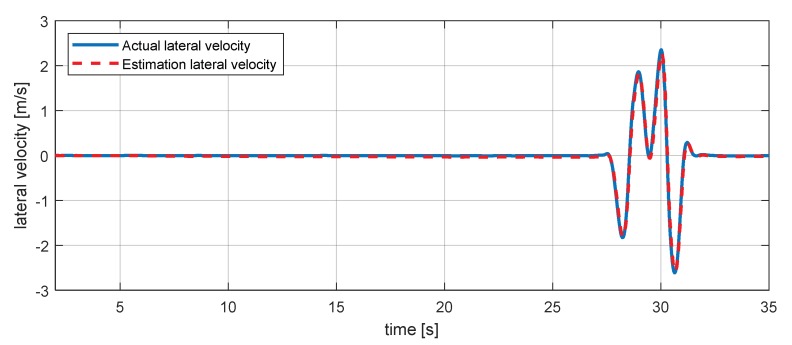
Estimation simulation result for lateral velocity.

Lateral velocity $\left(v_{y}\right)$ can be calculated using the lateral acceleration $\left(a_{y}\right)$ and the yaw rate $\left(\dot{\psi }\right)$ signal as shown in Equation (14) [31,32]. There is no lateral velocity sensor, but we used the equation to help understand the vehicle dynamics model used in residuals. For this reason, $v_{y}$ is also estimated using a dynamic model:

(14) $$v_{y}=\int_{t_{0}}^{t}\left(a_{y}-\dot{\psi }v_{x}\right)dt$$

and $v_{y}$ estimation simulation is also conducted as in Figure 8, where $a_{y}$ is the lateral acceleration of the vehicle.

The lateral velocity required for the calculation is calculated by Equation (14). Therefore, the residual equations for the fault diagnosis system are constructed by Equations (15) to (18).

(15) $$r_{1}:\;\omega_{fl}-\frac{{\hat{v}}_{fl}}{r}=\omega_{fl}-c_{1}\left(\dot{\psi },\;a_{y}\right)$$

(16) $$r_{2}:\;\omega_{fr}-\frac{{\hat{v}}_{fr}}{r}=\omega_{fr}-c_{2}\left(\dot{\psi },\;a_{y}\right)$$

(17) $$r_{3}:\;\omega_{rl}-\frac{{\hat{v}}_{rl}}{r}=\omega_{rl}-c_{3}\left(\dot{\psi }\right)$$

(18) $$r_{4}:\;\omega_{rr}-\frac{{\hat{v}}_{rr}}{r}=\omega_{rr}-c_{4}\left(\dot{\psi }\right)$$

For convenience of explanation, wheel speed $v_{fl,fr,\;rl,\;rr}$ is converted as wheel angular speed $\omega_{fl,fr,\;rl,\;rr}$ using tire radius r. The parameters semi-correlation table analysis was performed to analyze the relationship between the generated residual and the sensor signals. Table 1 shows the result of the parameters semi-correlation table analysis visualized as an X representation of the association between the sensors and the residual. 

Through analysis, residual 1 can easily be influenced by the yaw rate signal, the lateral acceleration signal, and the angular speed signal of the front left wheel. Likewise, residual 4 can be influenced by the yaw rate signal and the wheel angular speed of the rear right wheel. In other words, it is possible to analyze that residuals 1–4 are affected by different signals.

### 3.2. Steering Wheel Angle Residual

The steering angle has the constraint of Equation (19) and so constitutes Equation (20).

(19) $${\hat{\delta }}_{swa,1}=\frac{i_{r}l}{v_{x}}\left(1+\frac{v_{x}^{2}}{v_{ch}^{2}}\right)\frac{\omega_{fr}-\omega_{fl}}{l_{tw}}$$

(20) $${\hat{\delta }}_{swa,2}=\frac{i_{r}l}{v_{x}}\left(1+\frac{v_{x}^{2}}{v_{ch}^{2}}\right)\frac{\omega_{rr}-\omega_{rl}}{l_{tw}}$$

(21) $$r_{5}:\;\delta_{swa}-{\hat{\delta }}_{swa,1}=\delta_{swa}-c_{5}\left(\omega_{fl},\omega_{fr}\right)$$

(22) $$r_{6}:\;\delta_{swa}-{\hat{\delta }}_{swa,2}=\delta_{swa}-c_{6}\left(\omega_{rl},\omega_{rr}\right)$$

**Figure 9:**
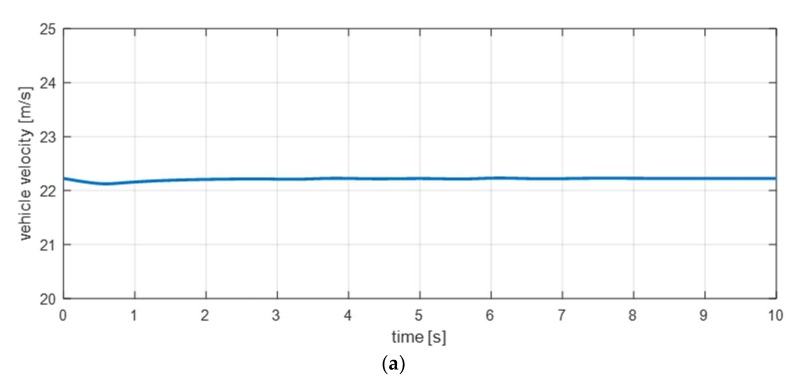

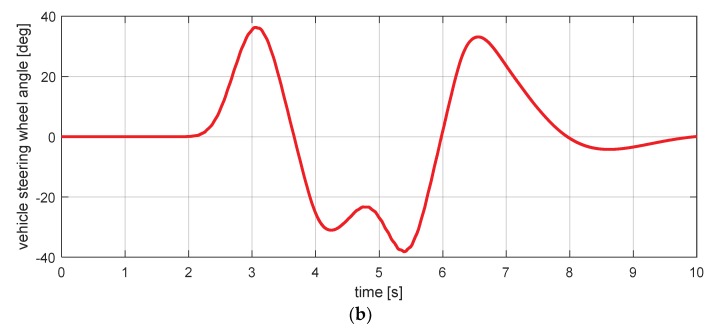
Estimation simulation scenario 2. (**a**) Vehicle speed; (**b**) vehicle steering wheel angle.

**Figure 10:**
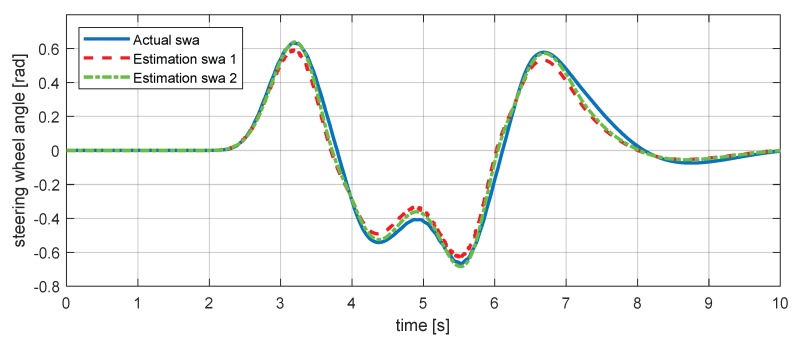
Estimation simulation result for the steering wheel angle.

Residuals 5 and 6, which were generated using Equations (19) and (20), are given in Equations (21) and (22) where $\delta_{swa}$ is the steering wheel angle, $i_{r}$ is the steering ratio, and $v_{ch}$ is the characteristic velocity of the vehicle [33,34]. For steering wheel angle estimation validation, we used scenario 2, shown in Figure 9. Figure 10 shows the validation simulation result of the sensor value with estimation values.

Table 2 shows the parameters semi-correlation table matrix result of residuals 5 and 6. Similar to Table 1, the signals used have a different influence on residuals 5 and 6.

### 3.3. Suspension Velocity Residual

To diagnose the failure of the vertical acceleration sensor installed in the vehicle, a normal force is calculated using the acceleration sensor signal. However, the normal force cannot be measured by sensors. Thus, to calculate the polynomial error method residual, the normal force was also calculated by considering the weight shift of the vehicle using the longitudinal and lateral acceleration sensor signals.

**Figure 11:**
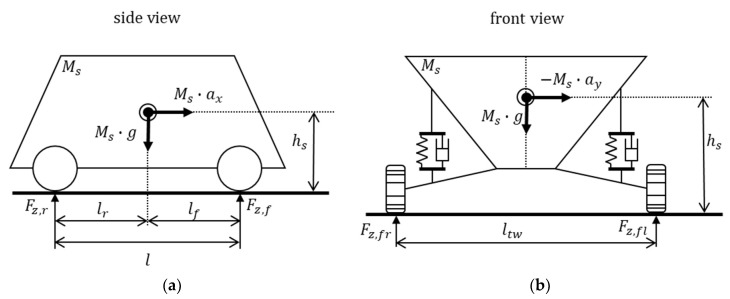
Vehicle normal force scheme. (**a**) Vehicle side view; (**b**) vehicle front view.

Figure 11a shows the vertical forces of the vehicle on the road from the side view. Figure 11b shows the vertical forces of the vehicle from the front view. The vertical force acting on each wheel can be calculated from Equations (23) to (26) by considering the weight shift according to the behavior of the vehicle when the longitudinal acceleration and lateral acceleration are known [35], where $f_{z,i}\left(i=fl,\;fr,\;rl,\;rr\right)$ is the normal force, $M_{s}$ is the vehicle mass, g is the gravitational acceleration, $l_{r}$ is the length of the rear wheel base, $h_{s}$ is the height from the ground to the mass center, and $a_{x}$ is the longitudinal acceleration of the vehicle.

(23) $$f_{z,fl,1}=\frac{M_{s}gl_{r}}{2l}-\frac{M_{s}a_{x}h_{s}}{2l}-\frac{M_{s}a_{y}h_{s}l_{r}}{l_{tw}l}$$

(24) $$f_{z,fr,1}=\frac{M_{s}gl_{r}}{2l}-\frac{M_{s}a_{x}h_{s}}{2l}+\frac{M_{s}a_{y}h_{s}l_{r}}{l_{tw}l}$$

(25) $$f_{z,rl,1}=\frac{M_{s}gl_{f}}{2l}+\frac{M_{s}a_{x}h_{s}}{2l}-\frac{M_{s}a_{y}h_{s}l_{f}}{l_{tw}l}$$

(26) $$f_{z,rr,1}=\frac{M_{s}gl_{f}}{2l}+\frac{M_{s}a_{x}h_{s}}{2l}+\frac{M_{s}a_{y}h_{s}l_{f}}{l_{tw}l}$$

**Figure 12:**
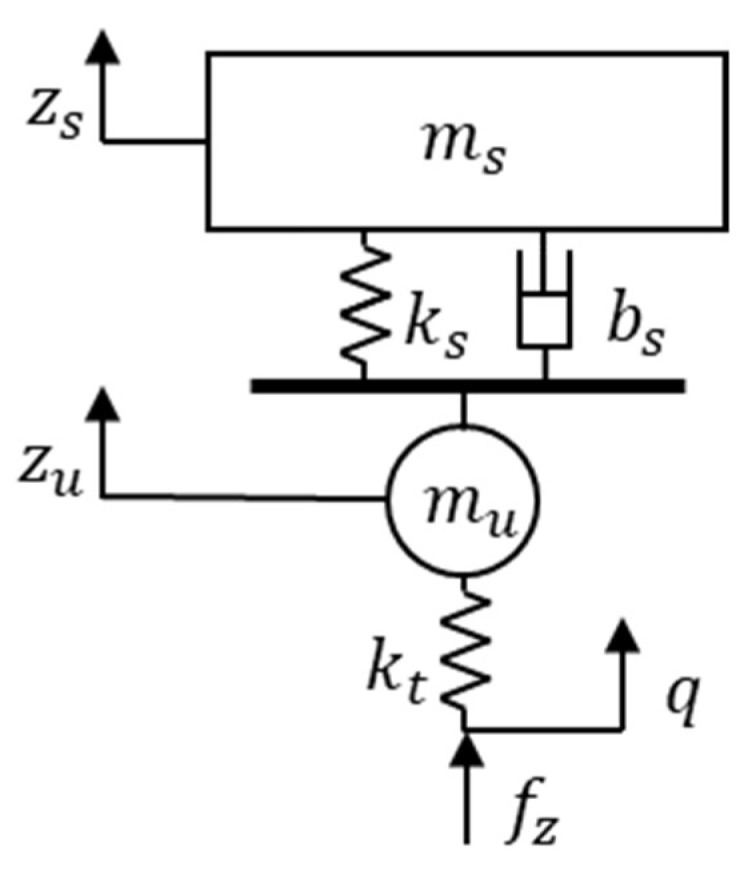
Quarter car model for normal force calculation.

To calculate the other normal force estimation, the quarter car model in Figure 12 was used [36,37].

(27) $$m_{s}\left({\ddot{z}}_{s}-g\right)+k_{s}\left(z_{s}-z_{u}\right)+b_{s}\left({\dot{z}}_{s}-{\dot{z}}_{u}\right)=0$$

(28) $$m_{s}\left({\ddot{z}}_{s}-g\right)+k_{s}\left(z_{s}-z_{u}\right)+b_{s}\left({\dot{z}}_{s}-{\dot{z}}_{u}\right)+k_{t}\left(z_{u}-q\right)=0$$

(29) $$f_{z}+k_{t}\left(q-z_{u}\right)=0$$

In the quarter car model, the normal force of each wheel has the relation of Equations (27) to (29), where $m_{s}$ is the sprung mass of the quarter car model, $m_{u}$ is the un-sprung mass of the quarter car model, $k_{s}$ is the suspension spring coefficient, $b_{s}$ is the suspension damper coefficient, $k_{t}$ is the tire spring coefficient, ${\ddot{z}}_{s}$ is the vertical acceleration of sprung mass, ${\dot{z}}_{s}-{\dot{z}}_{u}$ is the suspension velocity, $z_{s}-z_{u}$ is the suspension deflection, $z_{u}$ is the un-sprung mass height, q is the road profile, and $f_{z}$ is the normal force effect on the tire.

In this paper, assuming that the tire stiffness is ignored, the vertical force of each wheel is summarized by Equation (30).

(30) $$f_{z,i,2}=\left(m_{s,i}+m_{u,i}\right)g-m_{s,i}{\ddot{z}}_{s,i}-m_{u,i}{\ddot{z}}_{u,i}\;\left(i=fl,fr,rl,rr\right)$$

Therefore, residuals 7 to 10 for fault detection can be calculated by Equations (31) to (34).

(31) $$r_{7}:{\hat{f}}_{z,fl,1}-{\hat{f}}_{z,fl,2}=c_{7}\left(a_{x},\;a_{y},{\ddot{z}}_{s,fl},{\ddot{z}}_{u,fl}\right)$$

(32) $$r_{8}:{\hat{f}}_{z,fr,1}-{\hat{f}}_{z,fr,2}=c_{8}\left(a_{x},\;a_{y},{\ddot{z}}_{s,fr},{\ddot{z}}_{u,fr}\right)$$

(33) $$r_{9}:{\hat{f}}_{z,rl,1}-{\hat{f}}_{z,rl,2}=c_{9}\left(a_{x},\;a_{y},{\ddot{z}}_{s,rl},{\ddot{z}}_{u,rl}\right)$$

(34) $$r_{10}:{\hat{f}}_{z,rr,1}-{\hat{f}}_{z,rr,2}=c_{10}\left(a_{x},\;a_{y},{\ddot{z}}_{s,rr},{\ddot{z}}_{u,rr}\right)$$

**Figure 13:**
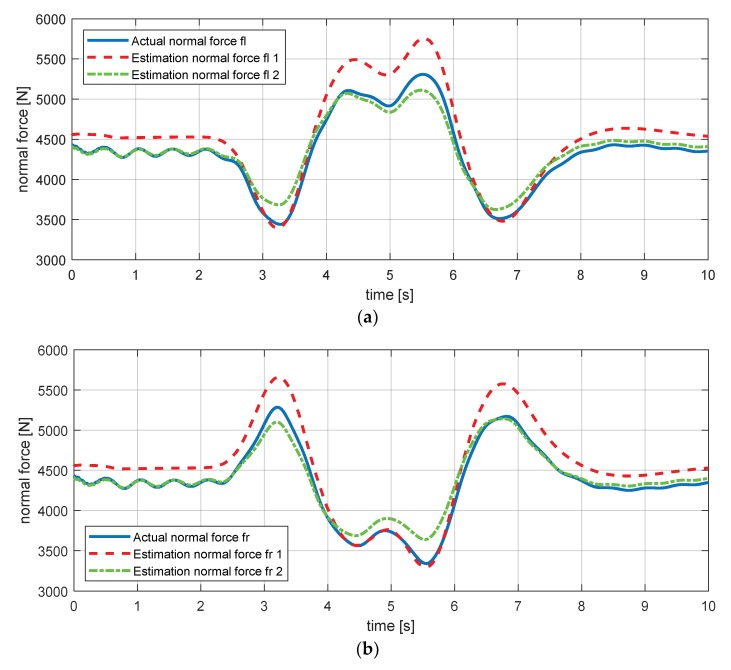

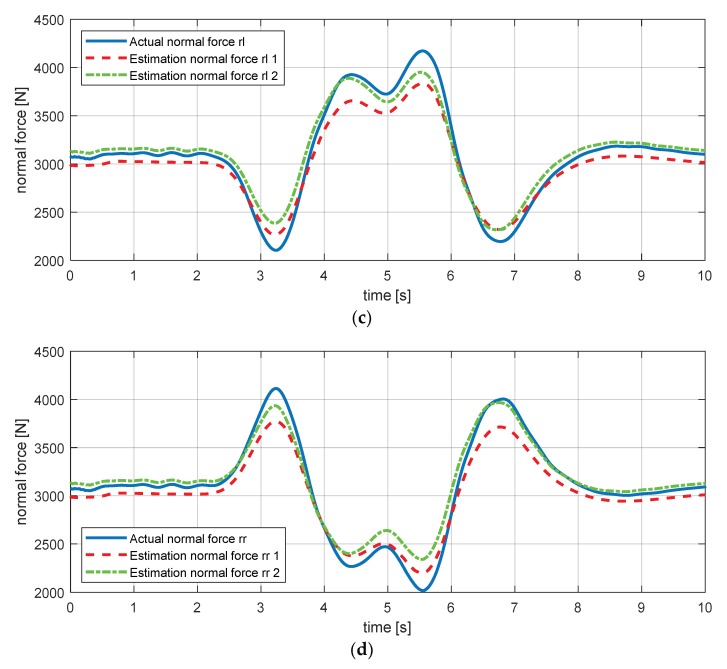
Estimation simulation result for the normal force. (**a**) Normal force—fl; (**b**) normal force—fr; (**c**) normal force—rl; (**d**) normal force—rr.

The simulation results in Figure 13 show that there are few errors, but the tendency of the estimation is the same. Since this error is due to the model uncertainty, it can be judged that it does not have a great influence on the failure judgment. 

However, the rear left, right wheel vertical acceleration (${\ddot{z}}_{u,rl},\;{\ddot{z}}_{u,rr}$), and rear left body acceleration (${\ddot{z}}_{s,rl}$) in Equations (31) to (34) are values calculated with existing vertical acceleration sensors as shown in Equations (35) to (36). In the sensitivity analysis, therefore, residuals 7 to 10 must be reconsidered as Equations (37) to (40).

(35) $${\dot{z}}_{s,fl}\left(t\right)=\int_{t_{0}}^{t}{\ddot{z}}_{s,fl}\left(t\right)dt\;{\dot{z}}_{s,fr}\left(t\right)=\int_{t_{0}}^{t}{\ddot{z}}_{s,fr}\left(t\right)dt\;{\dot{z}}_{s,rl}\left(t\right)=\int_{t_{0}}^{t}{\ddot{z}}_{s,fl}\left(t\right)dt-\int_{t_{0}}^{t}{\ddot{z}}_{s,fr}\left(t\right)dt+\int_{t_{0}}^{t}{\ddot{z}}_{s,rr}\left(t\right)dt\;{\dot{z}}_{s,rr}\left(t\right)=\int_{t_{0}}^{t}{\ddot{z}}_{s,rr}\left(t\right)dt$$

(36) $${\dot{z}}_{u,fl}\left(t\right)=\int_{t_{0}}^{t}{\ddot{z}}_{u,fl}\left(t\right)dt\;{\dot{z}}_{u,fr}\left(t\right)=\int_{t_{0}}^{t}{\ddot{z}}_{u,fr}\left(t\right)dt\;{\dot{z}}_{u,rl}\left(t\right)={\dot{z}}_{u,fl}\left(t+l/v_{x}\right)\;{\dot{z}}_{u,rr}\left(t\right)={\dot{z}}_{u,fr}\left(t+l/v_{x}\right)$$

The vertical body velocity is calculated by Equation (35), with the vertical wheel velocity calculated by Equation (36).

(37) $$r_{7}:{\hat{f}}_{z,fl,1}-{\hat{f}}_{z,fl,2}=c_{7}\left(a_{x},\;a_{y},\ddot{z_{s,fl}},\ddot{z_{u,fl}}\right)$$

(38) $$r_{8}:{\hat{f}}_{z,fr,1}-{\hat{f}}_{z,fr,2}=c_{8}\left(a_{x},\;a_{y},\ddot{z_{s,fr}},\ddot{z_{u,fr}}\right)$$

(39) $$r_{9}:{\hat{f}}_{z,rl,1}-{\hat{f}}_{z,rl,2}=c_{9}\left(a_{x},\;a_{y},\ddot{z_{s,fl}},\ddot{z_{s,fr}},\ddot{z_{s,rr}},\ddot{z_{u,fl}}\right)$$

(40) $$r_{10}:{\hat{f}}_{z,rr,1}-{\hat{f}}_{z,rr,2}=c_{10}\left(a_{x},\;a_{y},\ddot{z_{s,rr}},\ddot{z_{u,fr}}\right)$$

The normal force residual calculated with the existing sensors can be summarized by Equations (37) to (40). Therefore, the parameters semi-correlation table is expressed as in Table 3. However, unlike the previous parameters semi-correlation tables depicted in Table 1 and Table 2, the parameters semi-correlation table shows that the first and second columns tend to be the same, and that the third and sixth columns tend to be the same. This means that if the longitudinal acceleration sensor has a fault, residuals 7 to 10 make flags, but this cannot be distinguished from a lateral acceleration fault. Therefore, an additional residual using a signal described in Section 3.4 is essential for fault isolation.

### 3.4. Roll Angle Residual

Unlike the previously calculated residuals 1 to 6, residuals 7 to 10 are calculated by the polynomial error method instead of the output error method. This is because the estimated value used as a constraint is not known from the vehicle sensors. Similarly, since the roll angle of a car is not measured with a sensor, the roll-relevant residual is calculated in a polynomial error method using vehicle dynamics estimation.

The roll angle can be calculated from Equations (41) and (42), where $k_{roll}$ is the roll coefficient of the vehicle [38,39,40].

(41) $${\hat{\phi }}_{1}=\frac{\Delta z_{fl}-\Delta z_{fr}+\Delta z_{rl}-\Delta z_{rr}}{2l_{tw}}$$

(42) $${\hat{\phi }}_{2}=-\left(\frac{M_{s}h_{s}}{k_{roll}}\right)a_{y}$$

(43) $$\Delta z_{i}=z_{s,i}-z_{u,i}\;\left(i=fl,fr,rl,rr\right)$$

**Figure 14:**
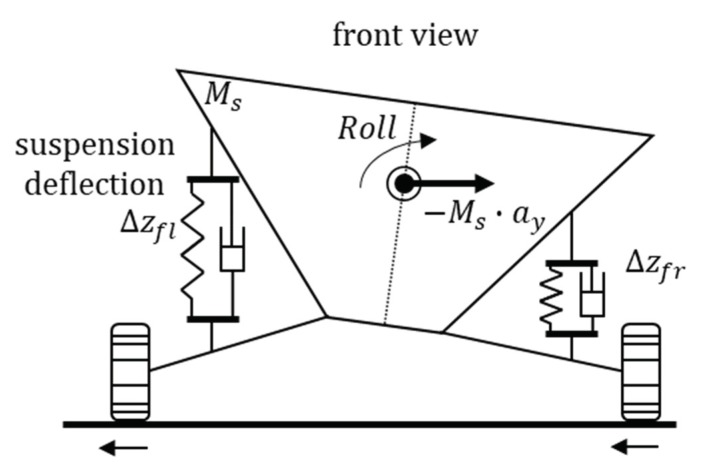
Vehicle roll angle scheme.

Figure 14 shows the roll angle scheme where $\Delta z_{i}\;\left(i=fl,\;fr,\;rl,\;rr\right)$ is presented as in Equation (43). Assuming a flat road surface, we can consider the suspension deflection as shown in Equation (43), substituting it into Equation (44).

(44) $$\Delta z_{i}=z_{s,i}\;\left(i=fl,fr,rl,rr\right)$$

**Figure 15:**
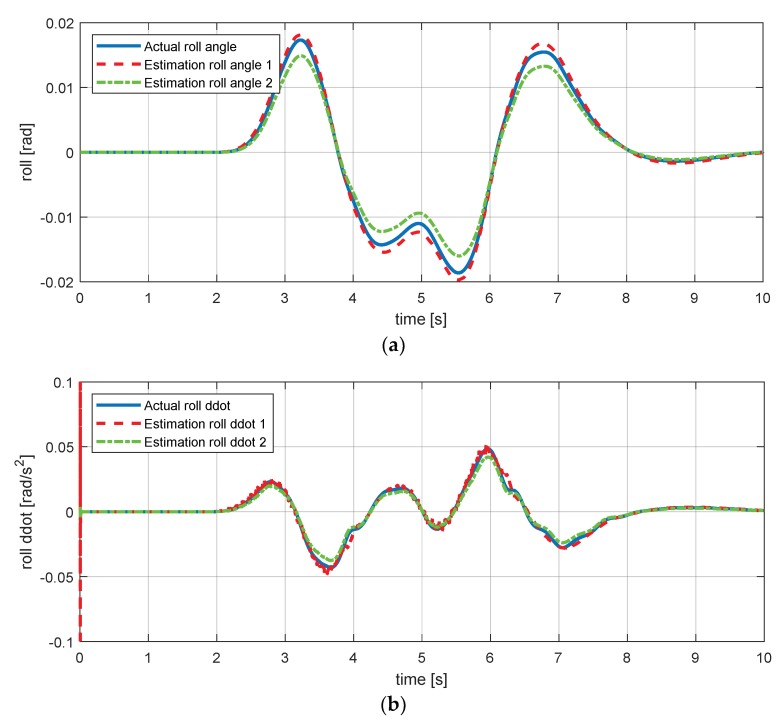
Estimation simulation result for the roll angle derivative of the roll rate. (**a**) Roll angle; (**b**) a derivative of the roll rate.

Moreover, Equation (42) presents another equation of the roll angle using the lateral acceleration signal. However, roll angle estimation formulas use only lateral acceleration signal and suspension deflection. This problem creates difficulty in a sensitivity analysis using partial derivatives. Therefore, the residual of estimation of the derivative of the roll rate was added as a constraint as in Equations (45) and (46). Figure 15 shows the result of the estimation simulations of the roll angle and the derivatives of the roll rate. Figure 15a,b both can confirmed that there is very little error.

(45) $$\ddot{{\hat{\phi }}_{1}}=\frac{{\ddot{z}}_{s,\;fl}-{\ddot{z}}_{s,fr}+{\ddot{z}}_{s,rl}-{\ddot{z}}_{s,rr}}{2l_{tw}}=\frac{{\ddot{z}}_{s,\;fl}-{\ddot{z}}_{s,fr}}{2l_{tw}}$$

(46) $$\ddot{{\hat{\phi }}_{2}}=-\left(\frac{M_{s}h_{s}}{k_{roll}}\right){\ddot{a}}_{y}$$

(47) $$r_{11}:\dot{{\hat{\phi }}_{1}}-\dot{{\hat{\phi }}_{2}}=c_{11}\left(a_{y},\ddot{z_{s,fl}},\ddot{z_{s,fr}}\right)$$

(48) $$r_{12}:\ddot{{\hat{\phi }}_{1}}-\ddot{{\hat{\phi }}_{1}}=c_{12}\left(a_{y},\ddot{z_{s,fl}},\ddot{z_{s,fr}}\right)$$

The parameters semi-correlation table of residuals 11 and 12 in Equations (47) and (48) is presented in Table 4. As shown in the parameters semi-correlation table, residual 11 is related to lateral acceleration, body vertical acceleration front left, and body vertical acceleration front right. However, for convenient sensitivity calculation, the sensitivity analysis of the residuals would be as shown in Table 5.

## 4. Residual Sensitivity Analysis

To develop the proposed FDI algorithms considering the residual sensitivity, a sensitivity analysis was conducted via the partial derivative. Each residual was partially differentiated by fault signals.

Table 6 presents the equations of residual sensitivity using the vehicle sensor signals. For the visualization of sensitivity, the simulation was conducted using scenario 1, which was presented in Section 3.1.

**Figure 16:**
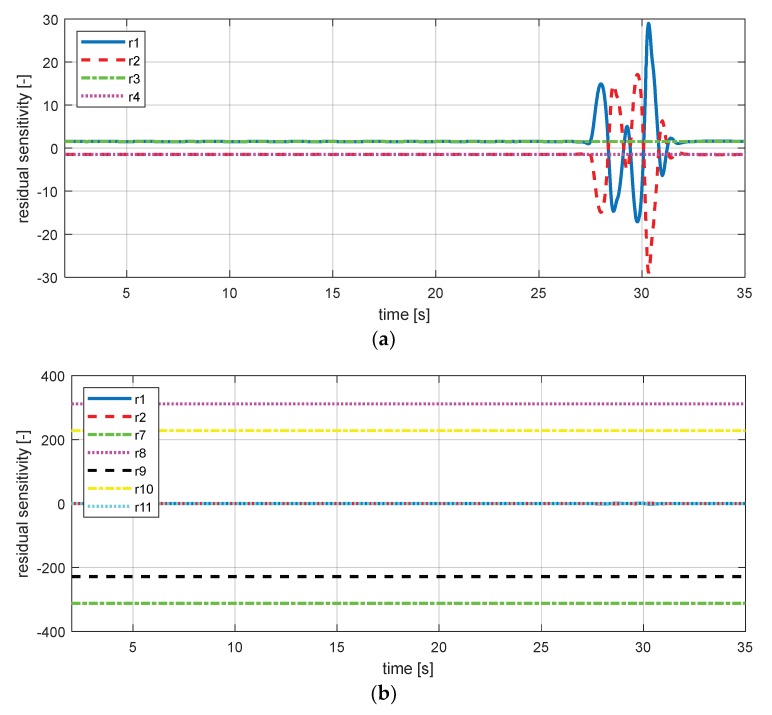

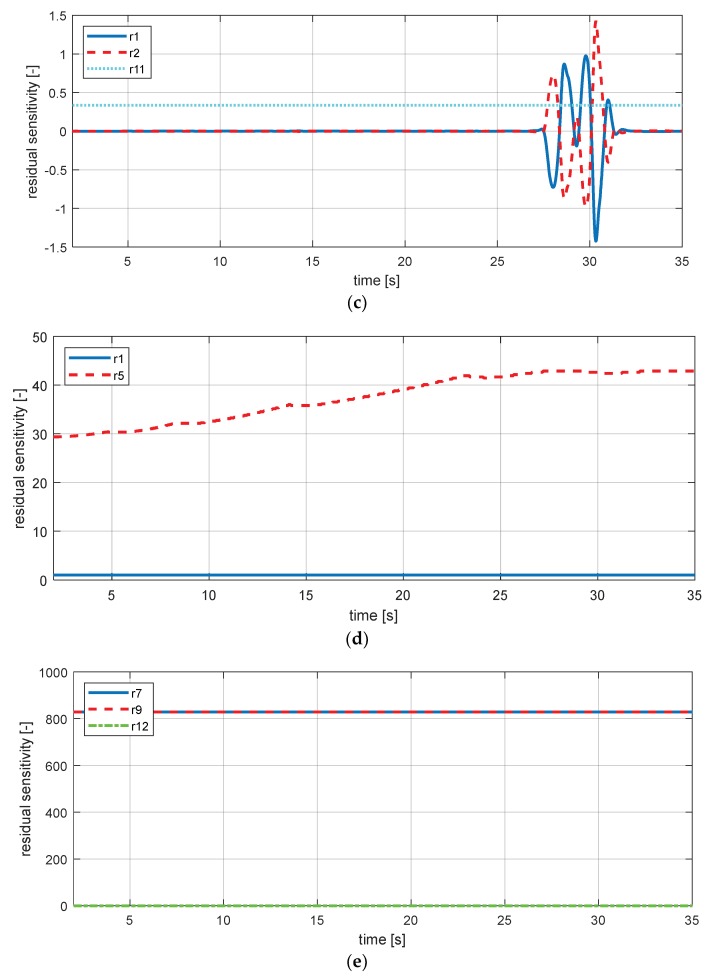
Sensitivity simulation result. (**a**) The sensitivity of yaw rate (residuals 1–4); (**b**) sensitivity of lateral acceleration (residuals 1, 2, 7, 8, 9, 10, 11); (**c**) sensitivity of lateral acceleration (residuals 1, 2, 11); (**d**) sensitivity of wheel angular speed—fl (residuals 1, 5); (**e**) sensitivity of body vertical acceleration—fl (residuals 1, 5).

Figure 16 shows the sensitivity of the residuals. In Figure 16a, residuals 1 to 4 are very sensitive to the yaw rate signal. However, in Figure 16b,c, residuals 1 and 2 have zero sensitivity for no lateral dynamic behavior. Also, the sensitivity of residual 1 to the yaw rate ($\frac{\partial r_{1}}{\partial \dot{\psi }}$) uses yaw as a rate signal. Therefore, threshold using these sensitivities are not appropriate for use in the fault detection; if the yaw rate sensor signal has a fault, the threshold for residual 1 also goes wrong. In the table of fault detection and isolation (Table 7), these results should be applied for accurate fault isolation. 

## 5. Fault Detection and Isolation Algorithm Test Result

**Figure 17:**
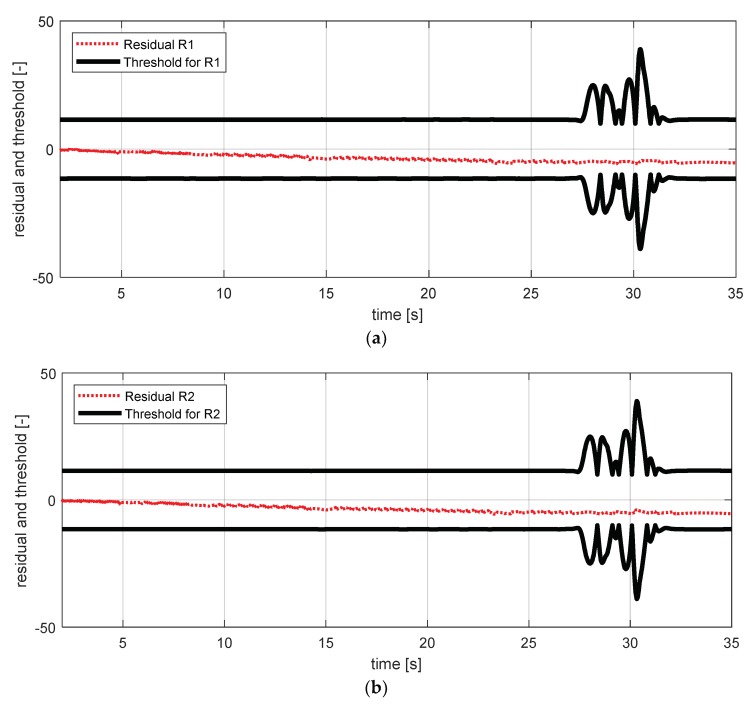

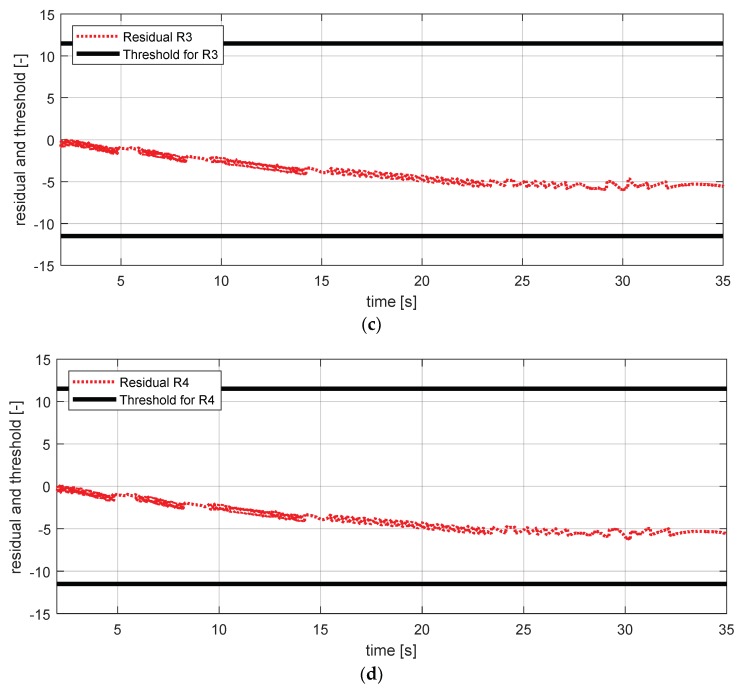
FDI (fault detection and isolation) simulation result for yaw rate sensor (normal). (**a**) Residual 1 and threshold; (**b**) residual 2 and threshold; (**c**) residual 3 and threshold; (**d**) residual 4 and threshold.

**Figure 18:**
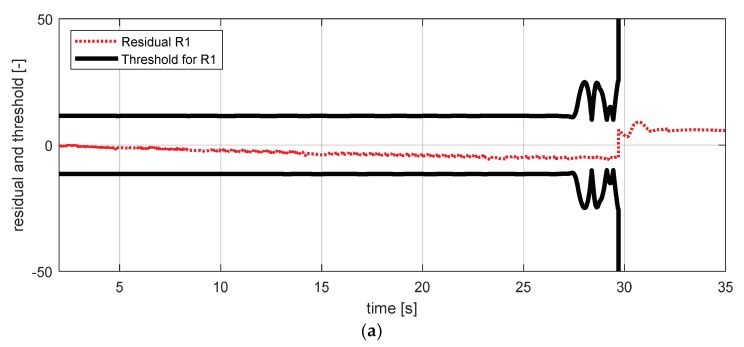

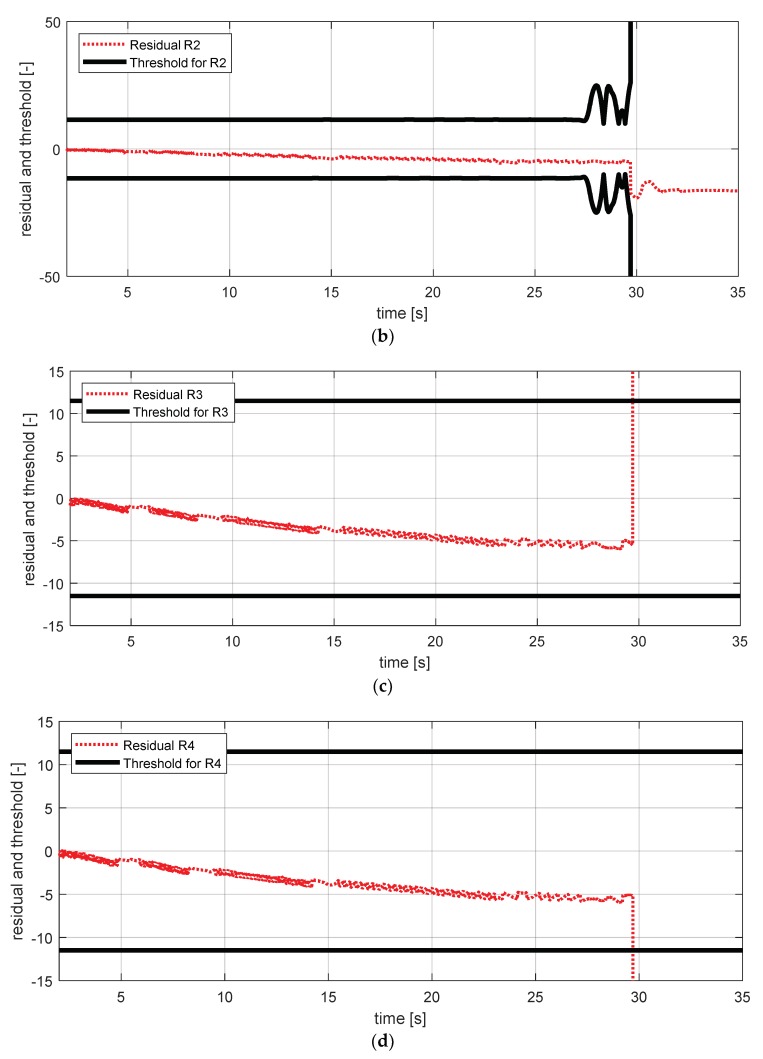
FDI simulation result for yaw rate sensor fault (fault). (**a**) Residual 1 and threshold; (**b**) residual 2 and threshold; (**c**) residual 3 and threshold; (**d**) residual 4 and threshold.

**Figure 19:**
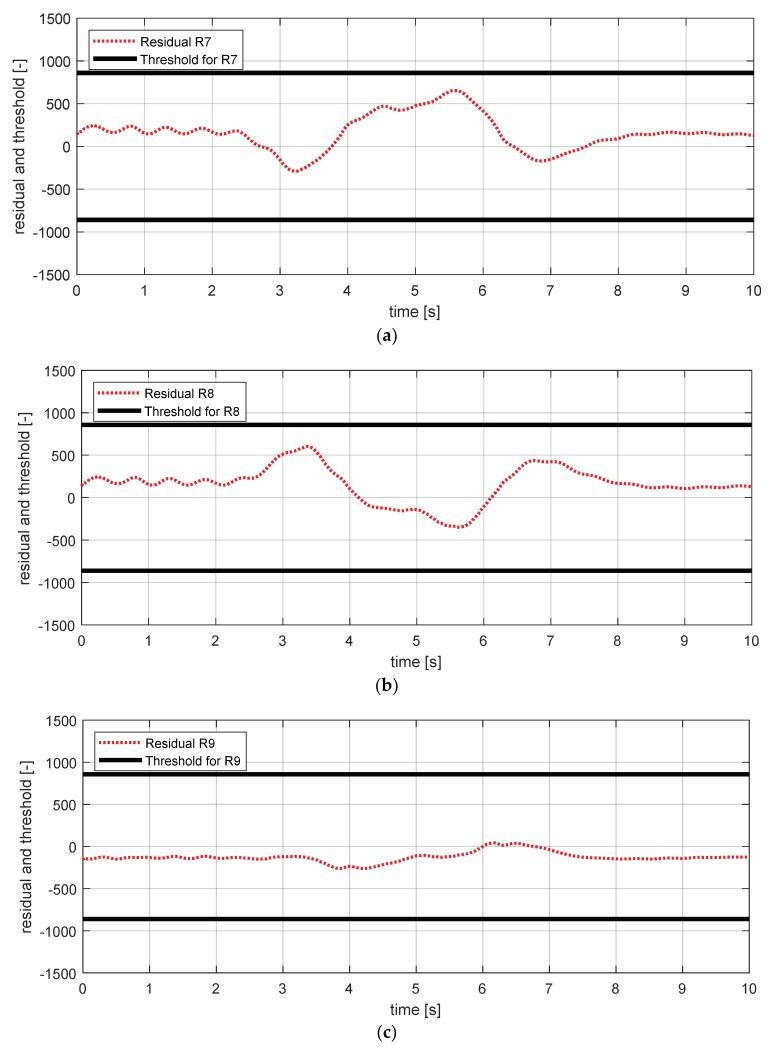

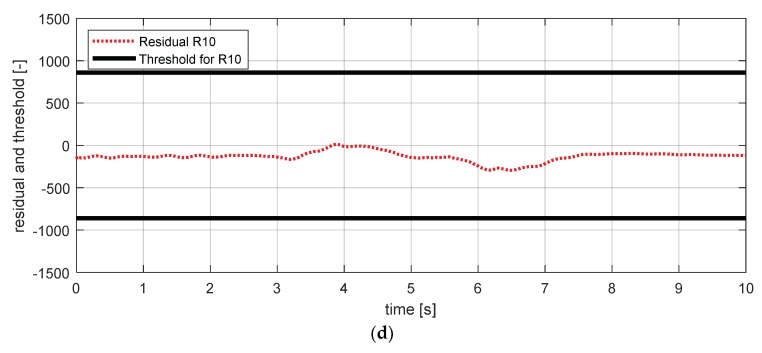
FDI simulation result for longitudinal acceleration sensor (normal). (**a**) Residual 7 and threshold; (**b**) residual 8 and threshold; (**c**) residual 9 and threshold; (**d**) residual 10 and threshold.

**Figure 20:**
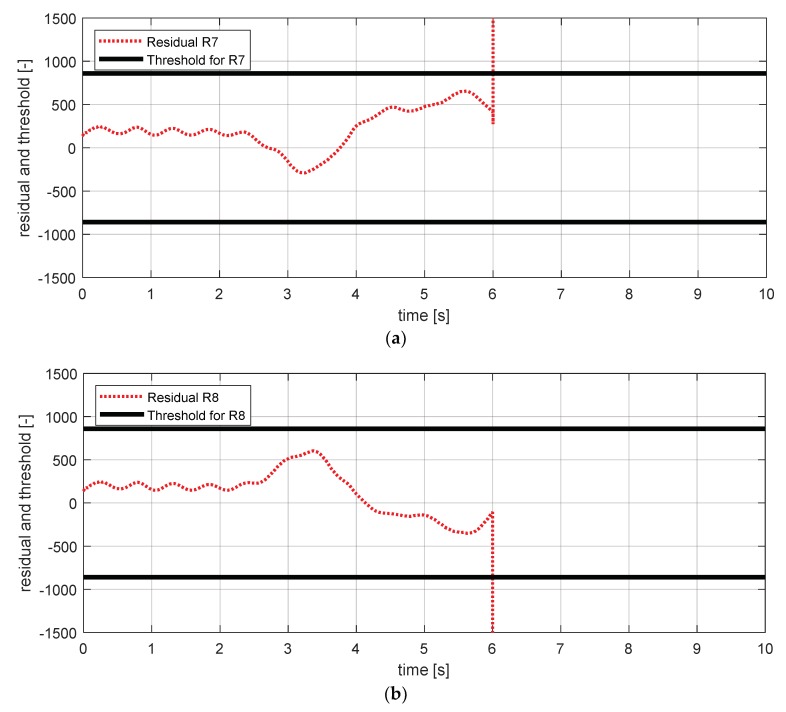

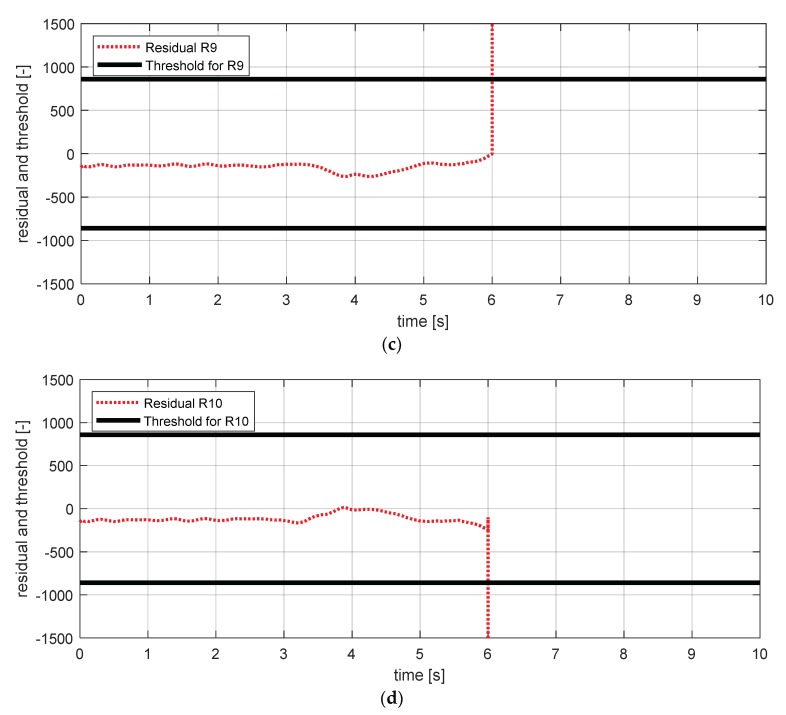
FDI simulation result for longitudinal acceleration sensor (fault). (**a**) Residual 7 and threshold; (**b**) residual 8 and threshold; (**c**) residual 9 and threshold; (**d**) residual 10 and threshold.

**Figure 21:**
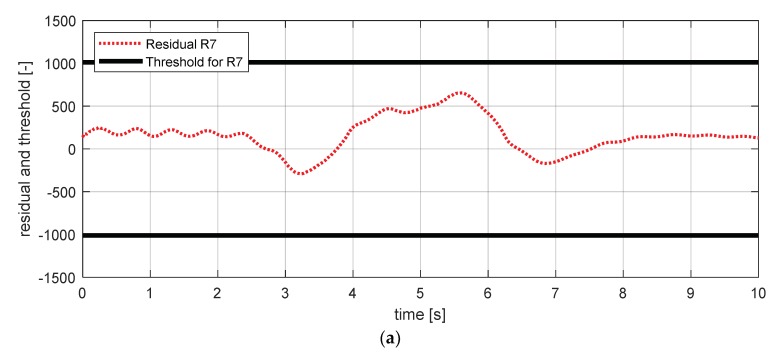

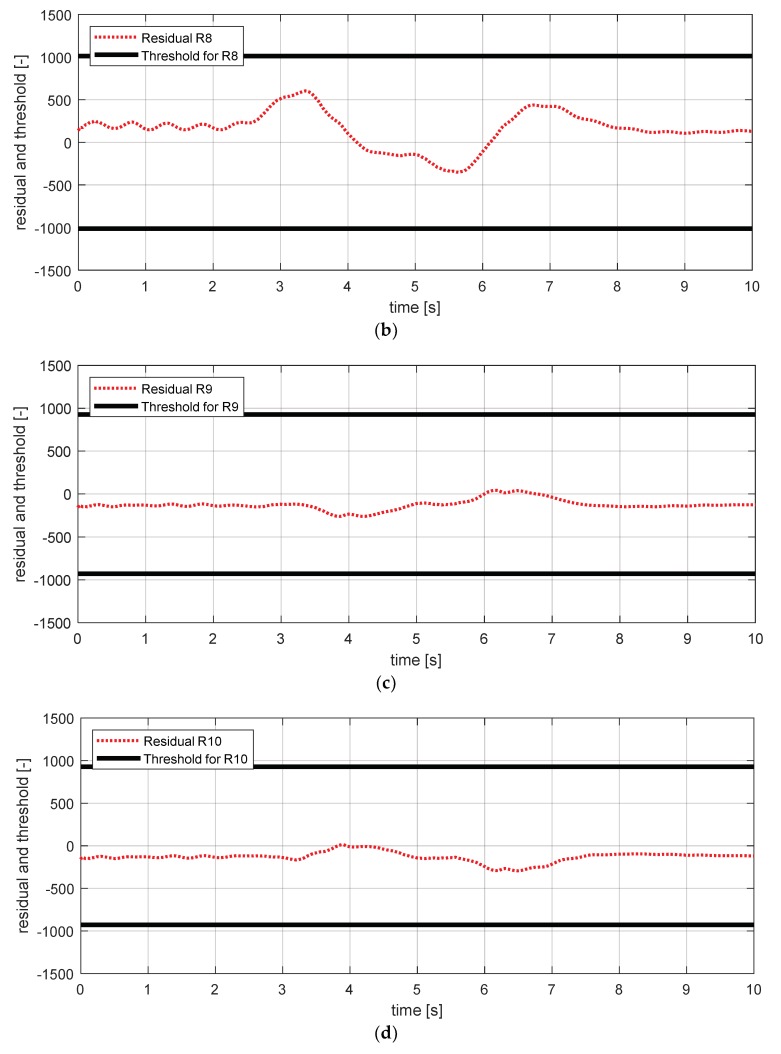

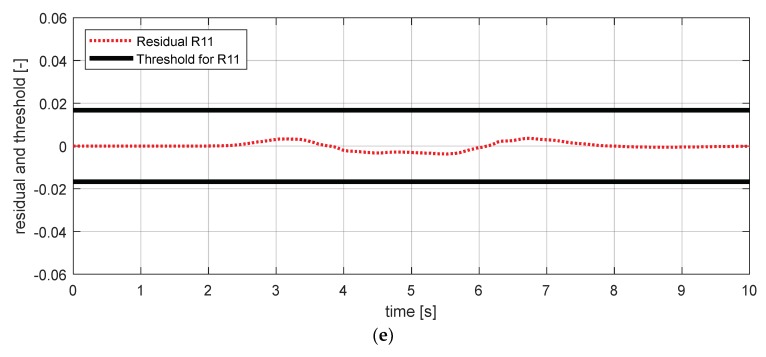
FDI simulation result for lateral acceleration sensor (normal). (**a**) Residual 7 and threshold; (**b**) residual 8 and threshold; (**c**) residual 9 and threshold; (**d**) residual 10 and threshold; (**e**) residual 11 and threshold.

**Figure 22:**
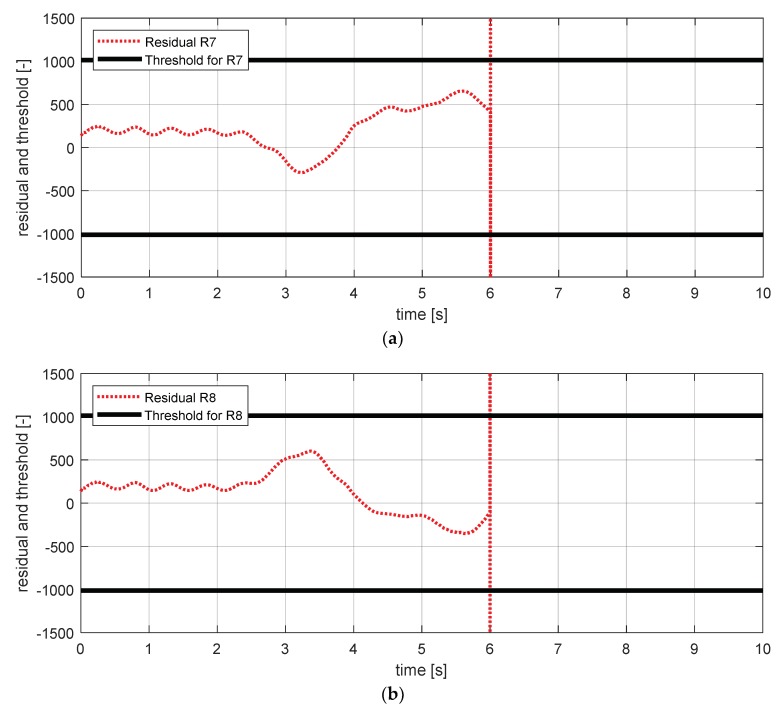

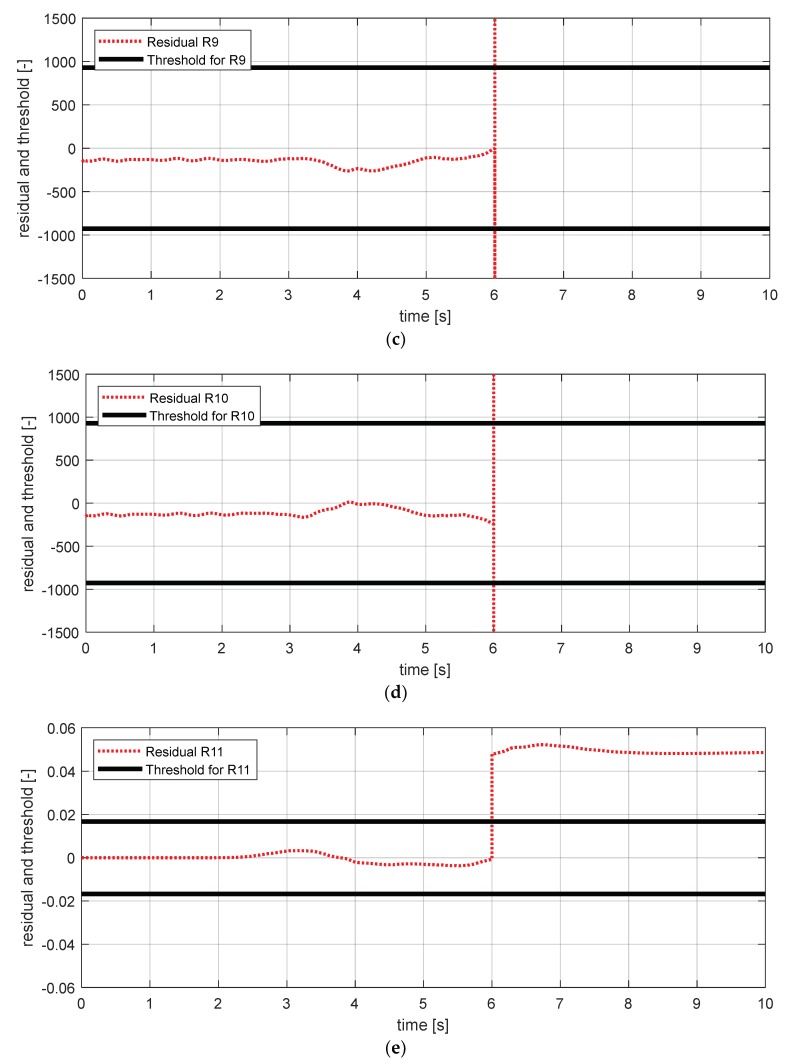
FDI simulation result for lateral acceleration sensor (fault). (**a**) Residual 7 and threshold; (**b**) residual 8 and threshold; (**c**) residual 9 and threshold; (**d**) residual 10 and threshold; (**e**) residual 11 and threshold.

**Figure 23:**
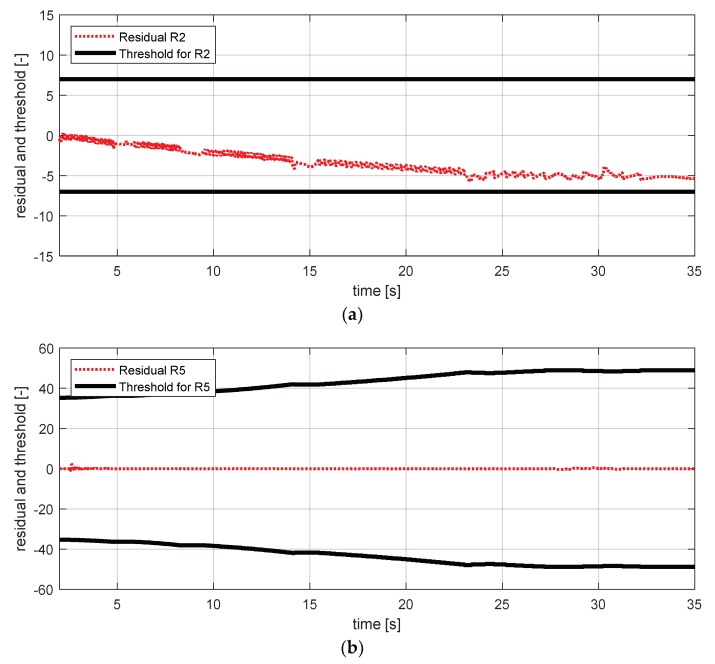
FDI simulation result for wheel angular speed sensor—fr (normal). (**a**) Residual 2 and threshold; (**b**) residual 5 and threshold.

**Figure 24:**
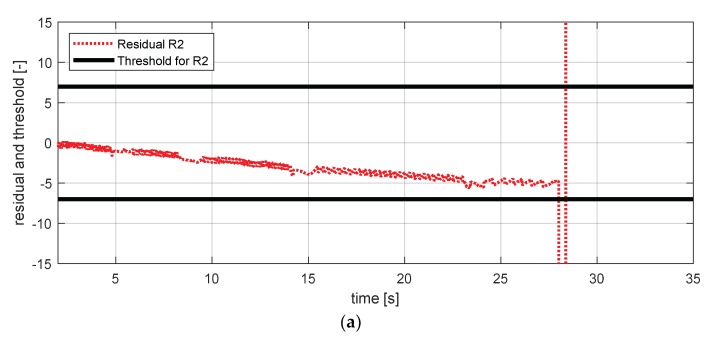

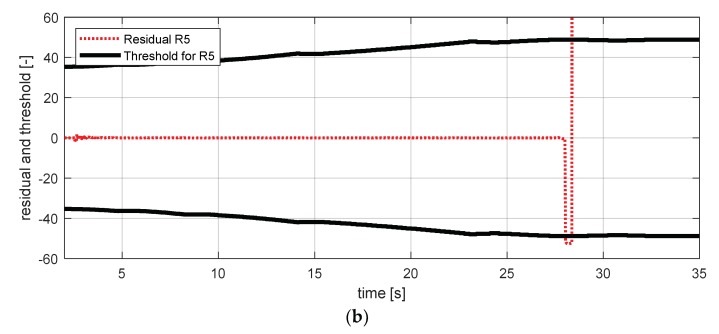
FDI simulation result for wheel angular speed sensor—fr (fault). (**a**) Residual 2 and threshold; (**b**) residual 5 and threshold.

**Figure 25:**
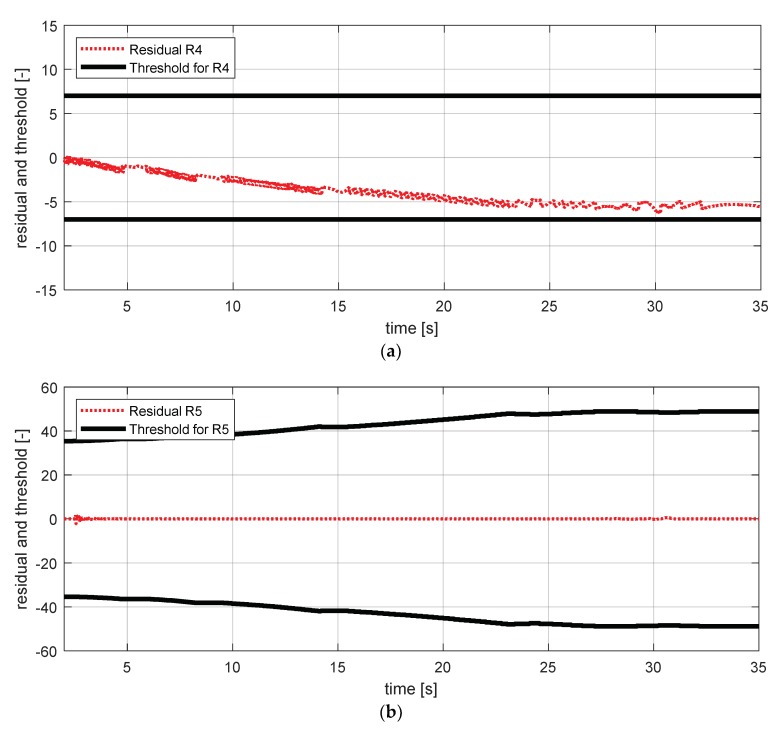
FDI simulation result for wheel angular speed sensor—rr (normal). (**a**) Residual 4 and threshold; (**b**) residual 5 and threshold.

**Figure 26:**
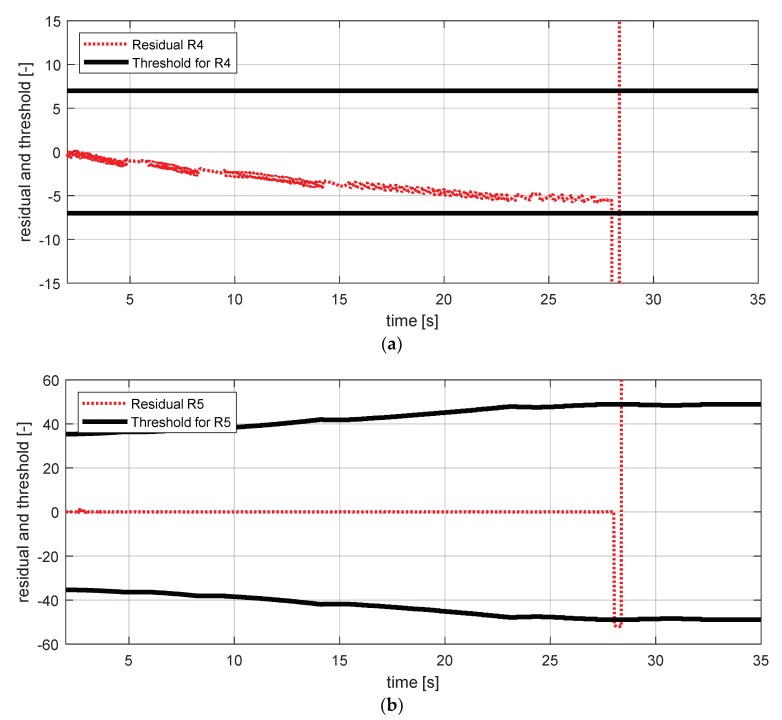
FDI simulation result for wheel angular speed sensor—rr (fault). (**a**) Residual 4 and threshold; (**b**) residual 5 and threshold.

**Figure 27:**
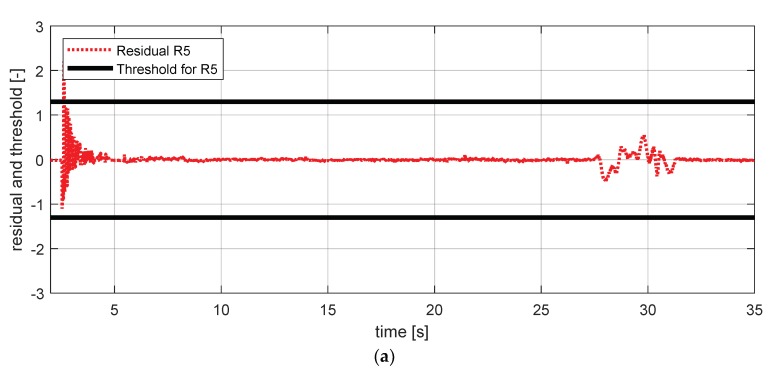

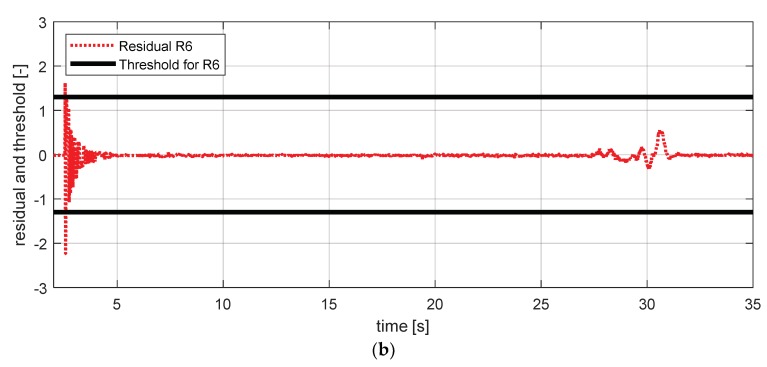
FDI simulation result for a steering wheel angle sensor (normal). (**a**) Residual 5 and threshold; (**b**) residual 6 and threshold.

**Figure 28:**
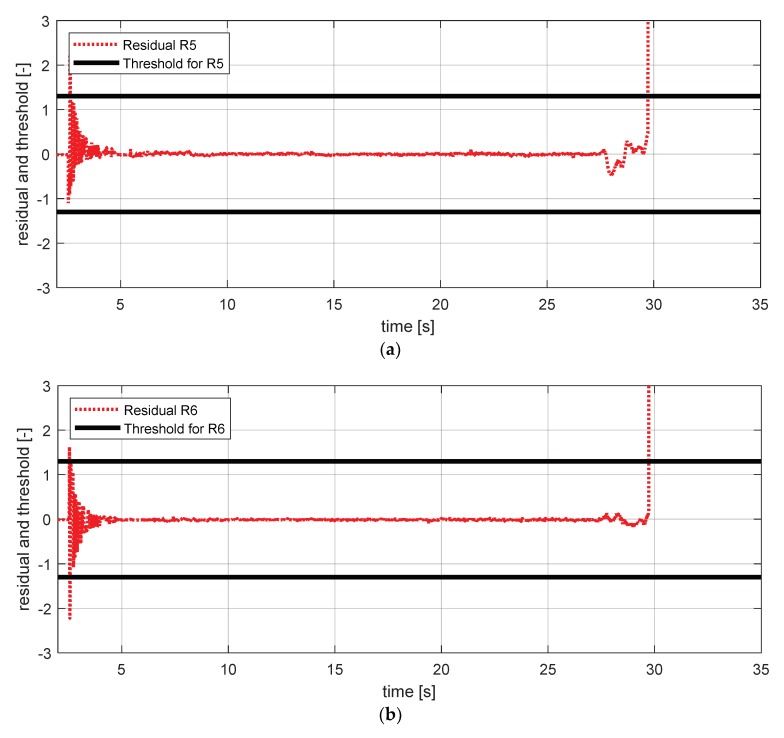
Simulation result for a steering wheel angle sensor (fault). (**a**) Residual 5 and threshold; (**b**) residual 6 and threshold.

**Figure 29:**
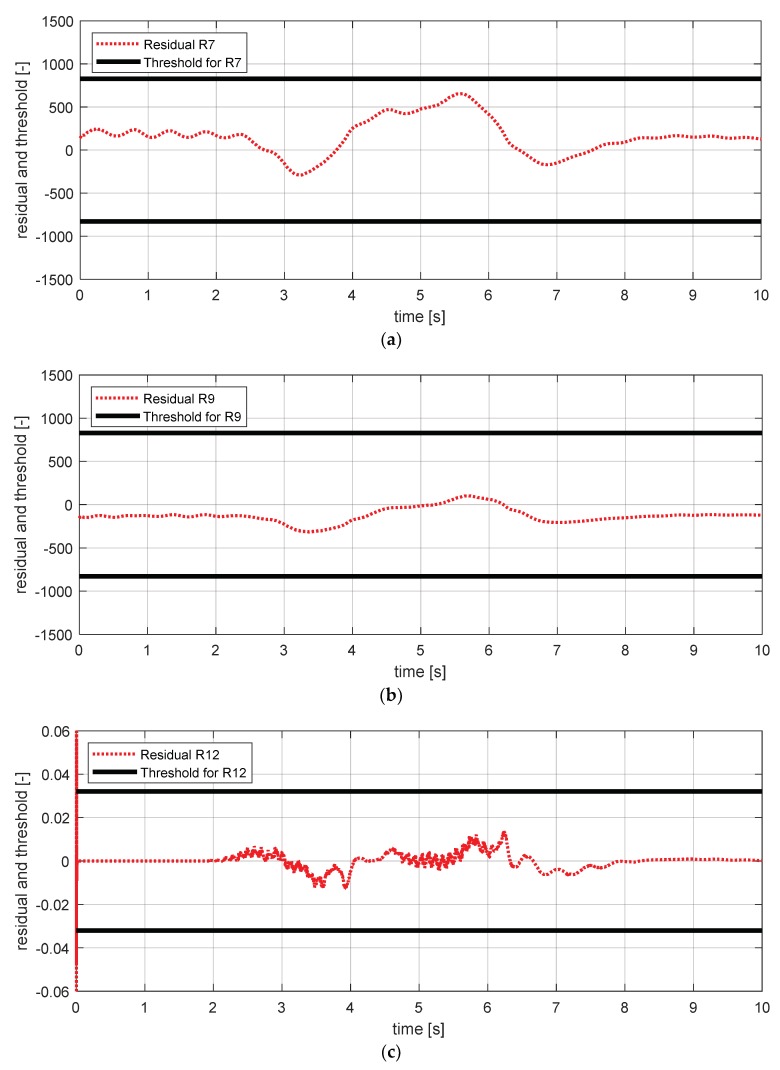
Simulation result for body vertical acceleration sensor—fl (normal). (**a**) Residual 7 and threshold; (**b**) residual 9 and threshold; (**c**) residual 12 and threshold.

**Figure 30:**
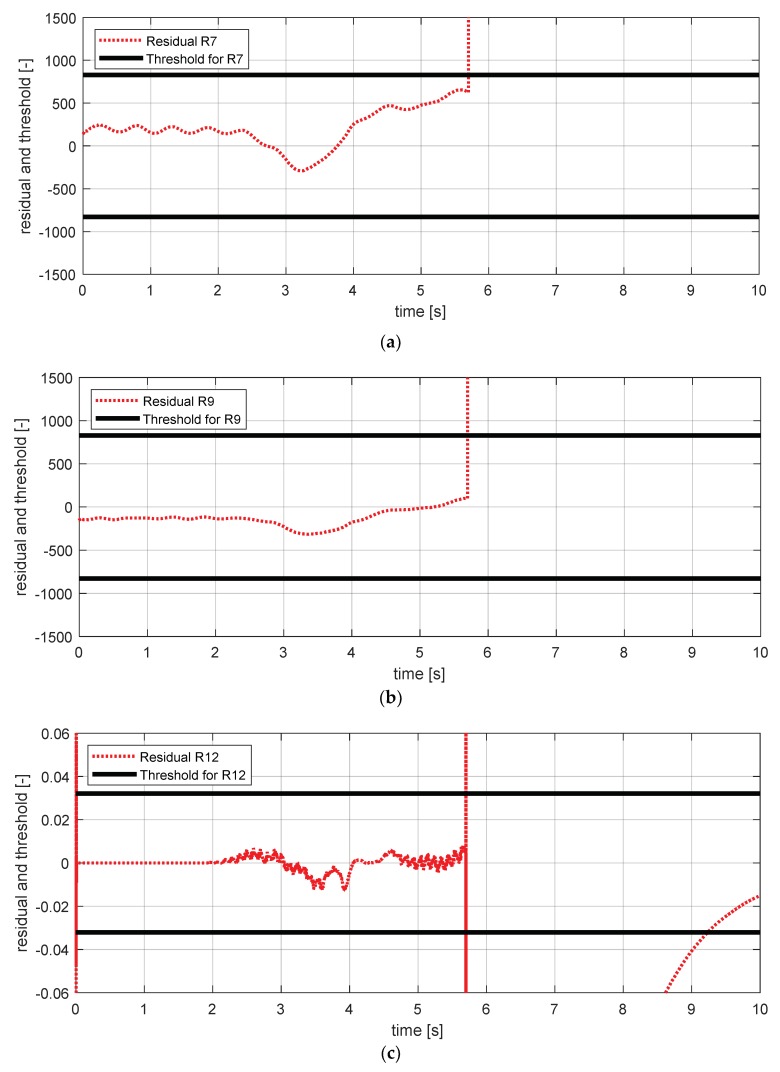
Simulation result for body vertical acceleration sensor—fl (fault). (**a**) Residual 7 and threshold; (**b**) residual 9 and threshold; (**c**) residual 12 and threshold.

**Figure 31:**
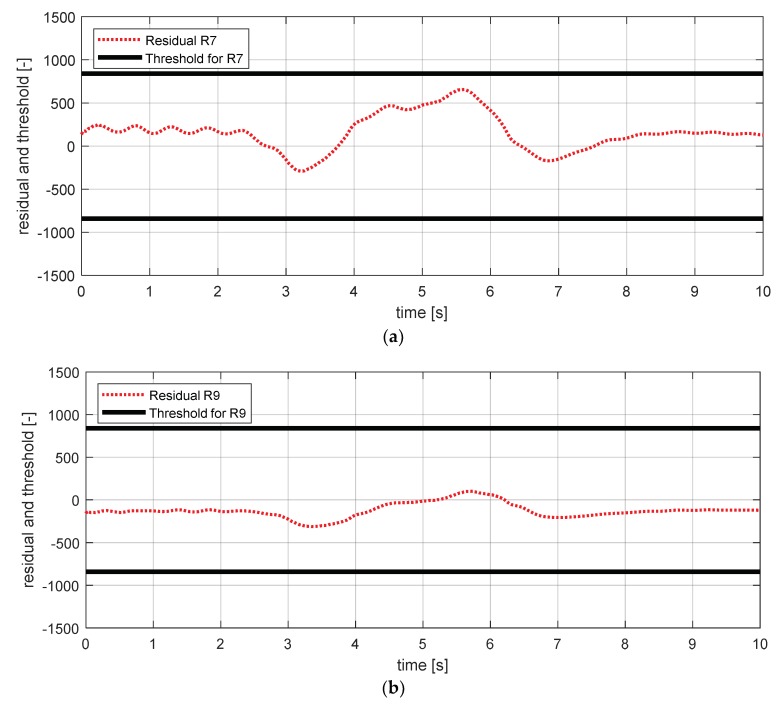
Simulation result for wheel vertical acceleration sensor—fl (normal). (**a**) Residual 7 and threshold; (**b**) residual 9 and threshold.

**Figure 32:**
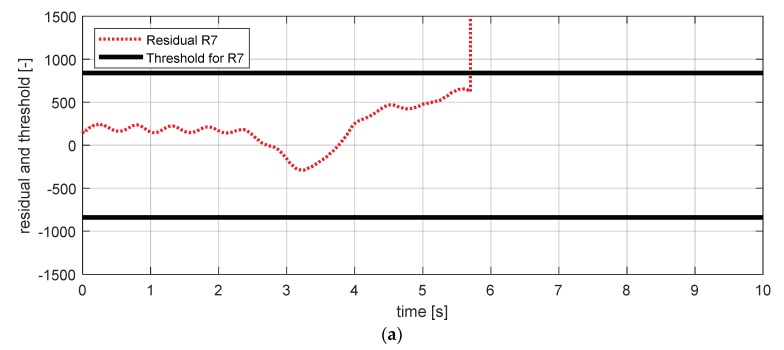

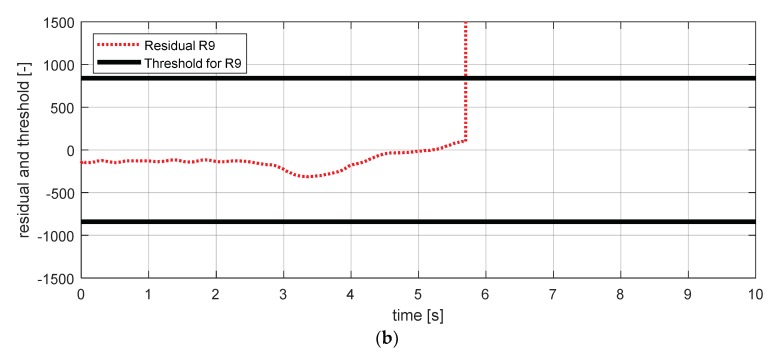
Simulation result for wheel vertical acceleration sensor—fl (fault). (**a**) Residual 7 and threshold; (**b**) residual 9 and threshold.

The proposed sensitivity-based FDI algorithm was verified by generating a faulty signal in an HILS-based simulation environment using RCP (rapid control prototyping) equipment implemented with a fault diagnosis algorithm. Using scenarios 1 and 2 in Figure 6 and Figure 9, each fault was injected, and it was validated that the fault can be detected by the sensitivity-applied threshold. Injected faults were also isolated as shown in Figure 17, Figure 18, Figure 19, Figure 20, Figure 21, Figure 22, Figure 23, Figure 24, Figure 25, Figure 26, Figure 27, Figure 28, Figure 29, Figure 30, Figure 31 and Figure 32. In these experiments, IPG’s Xpack4 and CarMaker in addition to dSPACE’s MicroAutoBox2 are used. Moreover, all signals used in the sensitivity-based FDI algorithm received by the CAN (controller area network) were connected to an HILS device. In order to ensure efficiency, only one experiment result was inserted for the same type of sensor fault result.

Figure 17 shows the experimental results in the normal situation with no fault and Figure 18 shows the simulation results in the case where the yaw rate sensor failed. In Figure 18, residuals 3 and 4 exceeded their thresholds, as designed by their sensitivities. On the other hand, as mentioned in Section 4, residuals 1 and 2 cannot detect a yaw rate sensor fault. This is because the threshold for residuals 1 and 2 use a faulty signal yaw rate signal (shown in Figure 18a,b).

Figure 19 shows the experimental results in the normal situation with no fault and Figure 20 shows the simulation results in the case where the longitudinal acceleration sensor failed. In Figure 20, residuals 7–10 exceeded their thresholds. This result is consistent with the expected results from the Table 7.

Figure 21 shows the experimental results in the normal situation with no fault and Figure 22 shows the simulation results in the case where the lateral acceleration sensor failed. In Figure 22, residuals 7–11 exceeded their thresholds, as designed by their sensitivities. As derived from Table 7 above, we reflected the failure sensitivity of the residual to the lateral acceleration sensor. In this process, residual 1 and 2 had a sensitivity of 0 when no steering occurred, as shown in Figure 16. For this reason, we decided not to use residuals 1 and 2 to detect lateral acceleration sensor faults. It was also confirmed that residual 11 effectively separates the failure of the longitudinal acceleration sensor and the failure of the lateral acceleration sensor.

Figure 23 shows the experimental results in the normal situation with no fault and Figure 24 shows the simulation results in the case where the wheel angular speed sensor at the front right failed. In Figure 24, residuals 2 and 5 exceeded their thresholds, as designed by their sensitivities. These results are consistent with the expected results from Table 7.

Figure 25 shows the experimental results in the normal situation with no fault and Figure 26 shows the simulation results in the case where the wheel angular speed sensor at the rear right failed. In Figure 26, residuals 4 and 5 exceeded their thresholds, as designed by their sensitivities. These results are consistent with the expected results from Table 7.

Figure 27 shows the experimental results in the normal situation with no fault and Figure 28 shows the simulation results in the case where the steering wheel angle sensor failed.

Figure 29 shows the experimental results in the normal situation with no fault and Figure 30 shows the simulation results in the case where the body vertical acceleration sensor at the front left failed. In Figure 30, residuals 7, 9, and 12 exceed their thresholds. In Section 3, residual 12 was added to separate the fault tendency of the body vertical acceleration front left from the fault tendency of wheel vertical acceleration front left. Figure 30 shows that residual 12 effectively detected the fault of the body vertical acceleration sensor at the front left in the HIL simulation.

Figure 31 shows the experimental results in the normal situation with no fault and Figure 32 shows the simulation results in the case where the wheel vertical acceleration sensor of front left failed. In this simulation result, it was confirmed that residuals 7 and 9 exceeded their threshold values, which is different from the results shown in Figure 30. Based on this, it was possible to separate the fault tendencies of the body vertical acceleration sensor at the front left from the wheel vertical acceleration sensor at the front left. This also showed that residual 12 effectively isolated the fault of the body and wheel vertical acceleration sensors at the front left in the HIL simulation.

## 6. Conclusions

In this paper, to diagnose the faults of road chassis vehicle sensors, sensitivity-based fault detection and an isolation algorithm were developed. The proposed algorithm was constructed based on the sensitivity of residuals and generated using the analytical method. To generate residuals, 12 vehicle dynamics equations were designed and used.

Since a large number of failures were diagnosed simultaneously, the scope of each residual was significantly different, causing difficulties in determining and separating the failures. To improve the accuracy of fault judgment, the sensitivity of the residuals was analyzed analytically and applied to a threshold. Moreover, to improve the accuracy of the fault isolation, the sensitivity of the residual was applied to the previously analyzed parameters semi-correlation table to derive the fault isolation table. The open-loop state observer and FDI algorithms used in this paper were validated through a vehicle dynamic simulator and via HIL simulations.

As shown in the simulation results in Figure 17, Figure 18, Figure 19, Figure 20, Figure 21, Figure 22, Figure 23, Figure 24, Figure 25, Figure 26, Figure 27, Figure 28, Figure 29, Figure 30, Figure 31 and Figure 32, the results of the fault detection conditions using residual sensitivities are similar to those obtained by the adaptive threshold method introduced in previous studies. However, the difference in this paper is that the sensitivity of each residual is analyzed to take into account the uncertainty of the model. This study will help researchers who study faults in sensor-equipped commercial vehicles as well as fault-tolerant controllers of autonomous vehicles that control vehicles with sensor information. Further research will consider developing FDI algorithms using a closed-loop based observer and its sensitivity.

## Figures and Tables

**Table 1:** Parameters semi-correlation table of wheel angular speed residuals.

	$\dot{\psi }$	$a_{y}$	$w_{fl}$	$w_{fr}$	$w_{rl}$	$w_{rr}$
$r_{1}$	X	X	X			
$r_{2}$	X	X		X		
$r_{3}$	X				X	
$r_{4}$	X					X

**Table 2:** Parameters semi-correlation table of steering wheel angle residuals.

	$w_{fl}$	$w_{fr}$	$w_{rl}$	$w_{rr}$	$\delta_{swa}$
$r_{5}$	X	X			X
$r_{6}$			X	X	X

**Table 3:** Parameters semi-correlation table of normal force residuals.

	$a_{x}$	$a_{y}$	${\ddot{z}}_{s,fl}$	${\ddot{z}}_{s,fr}$	${\ddot{z}}_{s,rr}$	${\ddot{z}}_{u,fl}$	${\ddot{z}}_{u,fr}$
$r_{7}$	X	X	X			X	
$r_{8}$	X	X		X			X
$r_{9}$	X	X	X	X	X	X	
$r_{10}$	X	X			X		X

**Table 4:** Parameters semi-correlation table of roll residuals.

	$a_{y}$	${\ddot{z}}_{s,fl}$	${\ddot{z}}_{s,fr}$
$r_{11}$	X	-	-
$r_{12}$	-	X	X

**Table 5:** Parameters semi-correlation table of all residuals.

	$\dot{\psi }$	$a_{x}$	$a_{y}$	$w_{fl}$	$w_{fr}$	$w_{rl}$	$w_{rr}$	$\delta_{swa}$	${\ddot{z}}_{s,fl}$	${\ddot{z}}_{s,fr}$	${\ddot{z}}_{s,rr}$	${\ddot{z}}_{u,fl}$	${\ddot{z}}_{u,fr}$
$r_{1}$	X		X	X									
$r_{2}$	X		X		X								
$r_{3}$	X					X							
$r_{4}$	X						X						
$r_{5}$				X	X			X					
$r_{6}$						X	X	X					
$r_{7}$		X	X						X			X	
$r_{8}$		X	X							X			X
$r_{9}$		X	X						X	X	X	X	
$r_{10}$		X	X								X		X
$r_{11}$			X						-	-			
$r_{12}$			-						X	X			

**Table 6:** The sensitivity of residuals with a fault signal.

Fault Signal	Sensitivity (Partial Derivative)
$\dot{\psi }$	$\frac{\partial r_{1}}{\partial \dot{\psi }}=\frac{1}{r}\left(\frac{l_{tw}}{2}-\frac{l_{f}v_{y}}{v_{x}}+\frac{\dot{\psi }l_{f}\int v_{x}dt}{v_{x}}\right)$
	$\frac{\partial r_{2}}{\partial \dot{\psi }}=-\frac{1}{r}\left(\frac{l_{tw}}{2}-\frac{l_{f}v_{y}}{v_{x}}+\frac{\dot{\psi }l_{f}\int v_{x}dt}{v_{x}}\right)$
	$\frac{\partial r_{3}}{\partial \dot{\psi }}=\frac{l_{tw}}{2r}$
	$\frac{\partial r_{4}}{\partial \dot{\psi }}=-\frac{l_{tw}}{2r}$
$a_{x}$	$\frac{\partial r_{7}}{\partial a_{x}}=-\frac{M_{s}h_{s}}{2l}$
	$\frac{\partial r_{8}}{\partial a_{x}}=-\frac{M_{s}h_{s}}{2l}$
	$\frac{\partial r_{9}}{\partial a_{x}}=\frac{M_{s}h_{s}}{2l}$
	$\frac{\partial r_{10}}{\partial a_{x}}=\frac{M_{s}h_{s}}{2l}$
$a_{y}$	$\frac{\partial r_{1}}{\partial a_{y}}=-\frac{\dot{\psi }l_{f}t}{rv_{x}}$
	$\frac{\partial r_{2}}{\partial a_{y}}=\frac{\dot{\psi }l_{f}t}{rv_{x}}$
	$\frac{\partial r_{7}}{\partial a_{y}}=-\frac{M_{s}h_{s}l_{r}}{l_{tw}l}$
	$\frac{\partial r_{8}}{\partial a_{y}}=\frac{M_{s}h_{s}l_{r}}{l_{tw}l}$
	$\frac{\partial r_{9}}{\partial a_{y}}=--\frac{M_{s}h_{s}l_{f}}{l_{tw}l}$
	$\frac{\partial r_{10}}{\partial a_{y}}=\frac{M_{s}h_{s}l_{f}}{l_{tw}l}$
	$\frac{\partial r_{11}}{\partial a_{y}}=\frac{M_{s}h_{s}}{k_{roll}}$
$\omega_{fl}$	$\frac{\partial r_{1}}{\partial \omega_{fl}}=1$
	$\frac{\partial r_{5}}{\partial \omega_{fl}}=\frac{li_{r}}{l_{tw}}\left(1+{\left(\frac{v_{x}}{v_{ch}}\right)}^{2}\right)$
$\omega_{fr}$	$\frac{\partial r_{2}}{\partial \omega_{fr}}=1$
	$\frac{\partial r_{5}}{\partial \omega_{fr}}=-\frac{li_{r}}{l_{tw}}\left(1+{\left(\frac{v_{x}}{v_{ch}}\right)}^{2}\right)$
$\omega_{rl}$	$\frac{\partial r_{3}}{\partial \omega_{rl}}=1$
	$\frac{\partial r_{6}}{\partial \omega_{rl}}=\frac{li_{r}}{l_{tw}}\left(1+{\left(\frac{v_{x}}{v_{ch}}\right)}^{2}\right)$
$\omega_{rr}$	$\frac{\partial r_{4}}{\partial \omega_{rr}}=1$
	$\frac{\partial r_{6}}{\partial \omega_{rr}}=-\frac{li_{r}}{l_{tw}}\left(1+{\left(\frac{v_{x}}{v_{ch}}\right)}^{2}\right)$
$\delta_{swa}$	$\frac{\partial r_{5}}{\partial \delta_{swa}}=1$
	$\frac{\partial r_{6}}{\partial \delta_{swa}}=1$
${\ddot{z}}_{s,fl}$	$\frac{\partial r_{7}}{\partial {\ddot{z}}_{s,fl}}=m_{s,fl}$
	$\frac{\partial r_{9}}{\partial {\ddot{z}}_{s,fl}}=m_{s,fl}$
	$\frac{\partial r_{12}}{\partial {\ddot{z}}_{s,fl}}=\frac{1}{2l_{tw}}$
${\ddot{z}}_{s,fr}$	$\frac{\partial r_{8}}{\partial {\ddot{z}}_{s,fr}}=m_{s,fr}$
	$\frac{\partial r_{9}}{\partial {\ddot{z}}_{s,fr}}=-m_{s,fr}$
	$\frac{\partial r_{12}}{\partial {\ddot{z}}_{s,fr}}=\frac{1}{2l_{tw}}$
${\ddot{z}}_{s,rr}$	$\frac{\partial r_{9}}{\partial {\ddot{z}}_{s,rr}}=m_{s,rr}$
	$\frac{\partial r_{10}}{\partial {\ddot{z}}_{s,rr}}=m_{s,rr}$
${\ddot{z}}_{u,fl}$	$\frac{\partial r_{7}}{\partial {\ddot{z}}_{u,fl}}=m_{u,fl}$
	$\frac{\partial r_{9}}{\partial {\ddot{z}}_{u,fl}}=m_{u,fl}$
${\ddot{z}}_{u,fr}$	$\frac{\partial r_{8}}{\partial {\ddot{z}}_{u,fr}}=m_{u,fr}$
	$\frac{\partial r_{10}}{\partial {\ddot{z}}_{u,fr}}=m_{u,fr}$

**Table 7:** Fault detection and isolation table.

	$\dot{\varphi }$	$a_{x}$	$a_{y}$	$w_{fl}$	$w_{fr}$	$w_{rl}$	$w_{rr}$	$\delta_{swa}$	${\ddot{z}}_{s,fl}$	${\ddot{z}}_{s,fr}$	${\ddot{z}}_{s,rr}$	${\ddot{z}}_{u,fl}$	${\ddot{z}}_{u,fr}$
$r_{1}$	-		-	X									
$r_{2}$	-		-		X								
$r_{3}$	X					X							
$r_{4}$	X						X						
$r_{5}$				X	X			X					
$r_{6}$						X	X	X					
$r_{7}$		X	X						X			X	
$r_{8}$		X	X							X			X
$r_{9}$		X	X						X	X	X	X	
$r_{10}$		X	X								X		X
$r_{11}$			X						-	-			
$r_{12}$			-						X	X			
